# Characterizing the Potato Growing Regions in India Using Meteorological Parameters

**DOI:** 10.3390/life12101619

**Published:** 2022-10-17

**Authors:** Vinay Bhardwaj, Shashi Rawat, Jagesh Tiwari, Salej Sood, Vijay Kumar Dua, Baljeet Singh, Mehi Lal, Vikas Mangal, PM Govindakrishnan

**Affiliations:** 1 ICAR-Central Potato Research Institute, Shimla 171001, India; 2 ICAR-Central Potato Research Institute, Modipuram 250110, India

**Keywords:** potato, agro-ecologies, multi-environment trials (MET), meteorological parameters, physiological days (P days), growing degree days

## Abstract

Currently, the multi-location testing of advanced hybrids in India is carried out at 25 centers under the All India Co-ordinated Research Project on Potato (AICRP-P), which is spread across the country. These centres have been chosen to represent different potato growing regions based on soil and agronomic features. However, the reliable deployment of the newly bred varieties in different regions requires a scientific delineation of potato growing zones with homogenous climates. The present study was undertaken to develop homogenous zones in the Indian sub-continent based on the environmental parameters of the potato growing season. A total of 1253 locations were identified across the country as having a plausible potato growing season of at least 70 days with suitable thermal limits. Six variables including five meteorological parameters including Physiological days (P days), Growing degree days (GDD), Mean daily temperature, Mean night temperature and Mean daily incident solar radiation, together with altitude as the sixth variable, were used for Agglomerative Hierarchical Clustering (AHC) and the Principal Component Analysis by Multidimensional Scaling (MDS) technique to derive identical classes. The thematic map of the classes was overlaid on potato growing districts of India using ArcGIS 9.1 software. The study clearly depicted that the clustering technique can effectively delineate the target population of environments (TPE) for potato genotypes performing well at different testing environments in India. The study also identifies target locations for future focus on breeding strategies, especially the high night temperature class having a large expanse in India. This is also vital in view of the impending climate change situation.

## 1. Introduction

Multi-environment trials (MET) are the backbone of plant breeding and hitherto much emphasis had been laid on testing advanced hybrids at as many locations as possible so as to represent the various ecologies in which the crop is grown. The classification of adaptation domains for different ecologies in which the crop is commonly grown i.e., tropical, sub-tropical, temperate, etc. has been reported to be imprecise [1]. Chenu [2] opined that, while screening for genotype performance in MET allows for genotype improvements for the tested environments, it may misrepresent the target population of environments (TPE) and thus lead to the release of a genotype that is poorly adapted to the TPE. Moreover, conducting a large number of METs is costly and time-consuming.

Regarding the analysis of MET data per se, there has recently been increased emphasis on the use of statistical techniques like AMMI and Biplot to study genotype and environment interaction [3,4,5], and also to select genotypes [3,6,7]. However, the statistical techniques have inherent lacuna in that the results are applicable only to the environments used in the study, and extrapolation may lead to errors. Therefore, methods like conducting grouping trial sites have been adopted which can be useful in designing field testing plans in plant breeding programmes, but may not ultimately tell us where genotypes can perform well because the sites represent only a limited number of point locations [8]. Therefore, linking individual trial sites to larger regions of which they are representative for better targeting varieties to environments where they are expected to perform well have been called for [8]. One of the earliest studies on characterizing the growing regions for potato was the agro-ecological characterization to define areas for potato production globally [9]. This study used a crop simulation model and local environmental data to simulate the potential growth duration, potential yield and water-limited yield of potato crops at many locations and directly relate to environmental conditions so as to increase the efficiency of the long and laborious process of experimentation. While this method of using the model and local climate to characterize the production environment is one option, another is to use environmental variables to characterize the environment. Xu [10], stated that the environmental factors, such as temperature, radiation, precipitation, wind, and water availability, determine where a plant can grow while others determine how a plant grows. He coined the term “Envirotyping” for these studies.

Cluster analysis is another unsupervised data mining technique to group regions with similar climates. Hargrove and Hoffman [11] used a multivariate clustering technique to characterize ecoregions. Jones et al. [12] using climatic and soil data in a cluster analysis mapped the Brazilian Cerrados and then generalized them into homogenous regions while Delli et al. [13] used a similar approach to map wheat agro-ecologies in Algeria. The classification of growing regions based on climate has also been extensively used to define wine-growing regions [14,15].

**Figure 1:**
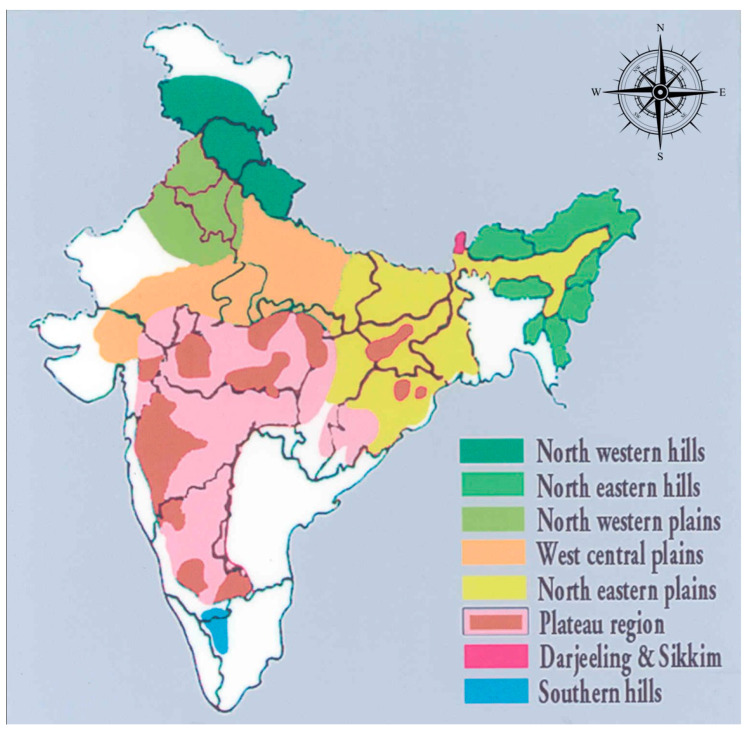
Popular Agro-climatic zones of India.

Potato has played a major role in the crop diversification and food security of India since its introduction about 400 years ago. It is an important cash crop and India ranks second in potato production in the world (52.59 million tonnes) after China, with an average productivity of 24.45 tonnes per hectare. The crop is grown in widely varying ecologies in the country, such as lowland plains, plateaus and hills. Seasonally it is grown during summers in the hills, autumn in the plains and winters in the warmer areas and climatically under long-day conditions in the hills and short-day conditions in the plains. On the basis of soil, climate and other agronomic features, the potato-growing regions in India have been grouped into eight zones. These are (i) the Northwestern plains (Punjab, Haryana and Rajasthan); (ii) the West-central plains (Uttar Pradesh and northwestern districts of Gujarat and Madhya Pradesh), (iii) North-eastern plains (Assam, Bihar, Jharkhand, West Bengal, Orissa, eastern Uttar Pradesh, and the northeastern districts of Madhya Pradesh and eastern Chhattisgarh), (iv) the plateau region (central and peninsular India comprising the states of Maharashtra, Karnataka, and parts of Gujarat); (v) the Northwestern hills (Jammu and Kashmir, Himachal Pradesh and Uttaranchal); (vi) the Northeastern hills (Northeastern Indian hills of Meghalaya, Manipur, Mizoram, Tripura, Nagaland and Arunachal Pradesh); (vii) the Southern hills (Nilgiris and Palini hills of Tamil Nadu), and (viii) the Sikkim and West Bengal hills (Darjeeling & Sikkim) (Figure 1) [16]. Nearly 85–90% of potato is grown in the vast Indo-Gangetic plains of north India during the winter season (Rabi) from October to March, while <5% of the area is cultivated in the Hills during the summer season from April to September/October. Furthermore, plateau regions of Southeastern, central and Peninsular India account for nearly 6% of the area where potato is grown as a rain-fed crop in Kharif season or an irrigated crop in the winter (Rabi) season [17].

Developing suitable varieties and good agricultural practices for such a huge area varying widely in environmental conditions and management practices is an onerous task. The present breeding strategy involves crossing of potato plants in the hills and the initial evaluation of the progenies at a few regional centers of the ICAR-Central Potato Research Institute, Shimla, India. The elite clones are then evaluated extensively in MET at 25 centers across the country through the All India Coordinated Research Project (AICRP) on Potato. The 25 centers have been categorized as northern plains, central plains, eastern plains, plateau and hills, etc. on the basis of geographic and physiographic features. However, as discussed earlier, there is a need for a scientific delineation of zones with similar climates and for the assigning of different testing centers to different regions so that target domains for hybrids performing well at a centre or group of centers can be identified realistically. Hence, this study was undertaken to develop homogenous zones based on the environmental parameters of the potato growing season.

## 2. Materials and Methods

Daily weather data from a large number of locations in India was generated using the MARKSIM weather generator “http://ccafs-climate.org/ (accessed on 7 October 2022)”. The plausible potato growing season at each location was delineated based on thermal limits including a maximum temperature less than 35 °C and a minimum temperature between 2 °C and 21 °C, at least three weeks after the maximum temperature condition is met. The locations with an estimated growing period of at least 70 days were selected, and in total 1253 locations were identified (Supplementary Table S1). From the meteorological data, the Physiological days (P days), Growing Degree Days, Mean temperature, Mean Night temperature and Mean daily incident solar radiation was calculated for the estimated growing period subject to the maximum of 120 days. The Physiological days (P days) accumulated during the thermally suitable period were calculated as per Sands et al. [18] using minimum, optimum and maximum cardinal temperatures of 7, 21 and 30 °C, respectively. The Growing Degree Days (GDD) were estimated using the average minus base temperature of 4 °C. The mean daily radiation and the mean temperature of the thermally suitable period were also calculated. The mean night temperature of the corresponding period was also calculated as per the formula given by Li [19].

A database of the derived meteorological parameters of each location along with the altitude and geo-coordinates of the location was developed. Cluster analysis of this database was carried out in XLSTAT. First, Agglomerative Hierarchical Clustering (AHC) was performed and then the Euclidean distance was estimated to create a distance cluster tree/dendrogram. Principal component analysis was thereafter performed by the Multidimensional Scaling (MDS) approach. Furthermore, a thematic map of the classes was prepared by interpolating the class numbers and reclassifying those using ArcGIS 9.1 software. The major nine classes were depicted on the map since the number of locations in the other six classes were too few to be visible on the map.

## 3. Results

Cluster-wise mean and range values of the meteorological parameters are presented in Table 1. The altitude of the different locations ranged from 2 to 2311 m above mean sea level (AMSL), while the mean daily temperature and mean night temperature of the growing season ranged from 7.33 to 26.02 °C and 11.26 to 24.31 °C, respectively. The differences in the temperature values were also reflected in the temperature-derived variables, such as GDD and P days, which ranged from 823.15 to 2367.9 and 352.9 to 1127.02, respectively. A similar variation was also observed in the mean daily incident radiation which ranged from 5084.39 to 24,968.04 KJ/m^2^/day.

**Figure 2:**
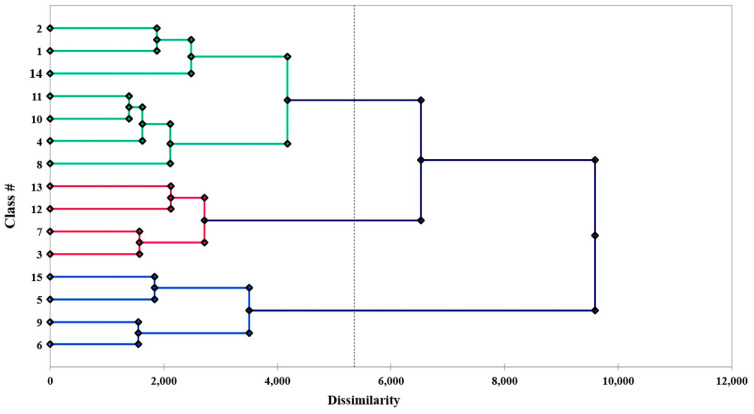
Dendrogram showing 15 classes representing different potato-growing locations in India.

**Figure 3:**
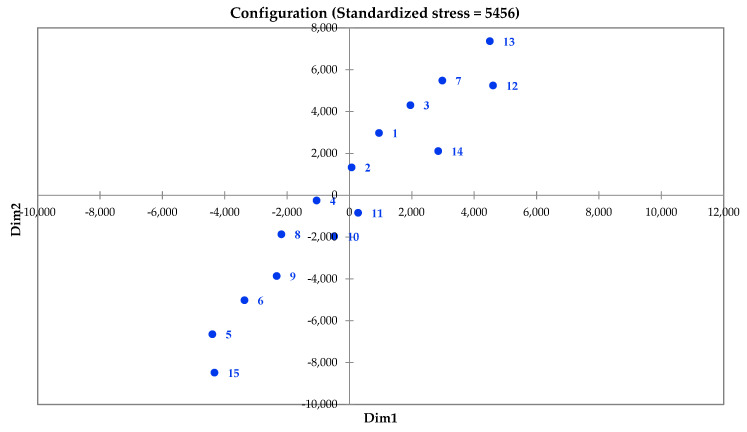
Two-dimensional Principal Component Analysis of the 15 classes representing different potato growing locations in India.

The clustering of the locations on the basis of the six variables including altitude, physiological days, growing degree days, mean night temperature, mean daily temperature, and mean radiation using the Agglomerative Hierarchical classification technique showed that the identified locations could be grouped into 15 classes (Figure 2). The results also showed that out of the 15 classes, the largest one is class 3 with 540 locations, followed by class 1 and class 7 with 399 and 166 locations, respectively (Table 1). The dissimilarity analysis showed that the Euclidean distance method was better than Pearson’s method since the variance decomposition in the former was 94.35% between classes and 5.65% within the class, while in the case of the latter it was 59.11 and 40.89%, respectively. A principal component analysis was performed using the Multidimensional Scaling (MDS) approach, and 15 classes were plotted onto different components, as shown in Figure 3.

The study also showed (Table 2) that the AICRP centers in the main potato-growing region of the Indo Gangetic plains including Faizabad, Kota, Kanpur, Hisar, Modipuram and Patna have been grouped together in class 1, with the only exception being Hassan, which has also been grouped in this class although geographically it is in Southern India. The centers experiencing warmer climates during the growing season, such as Deesa, Gwalior, Chhindwara, Bhubaneswar and Raipur, have been grouped together under class 3. The centers in eastern India viz Jorhat, Kalyani and Passighat have been grouped together in class 8, with the exception being Pantnagar. The Dharwad and Pune centers in the plateau have been grouped together in class 7 while the rest of the centers including Jalandhar, Ooty, Shillong, Srinagar and Shimla have been categorized into separate classes.

**Figure 4:**
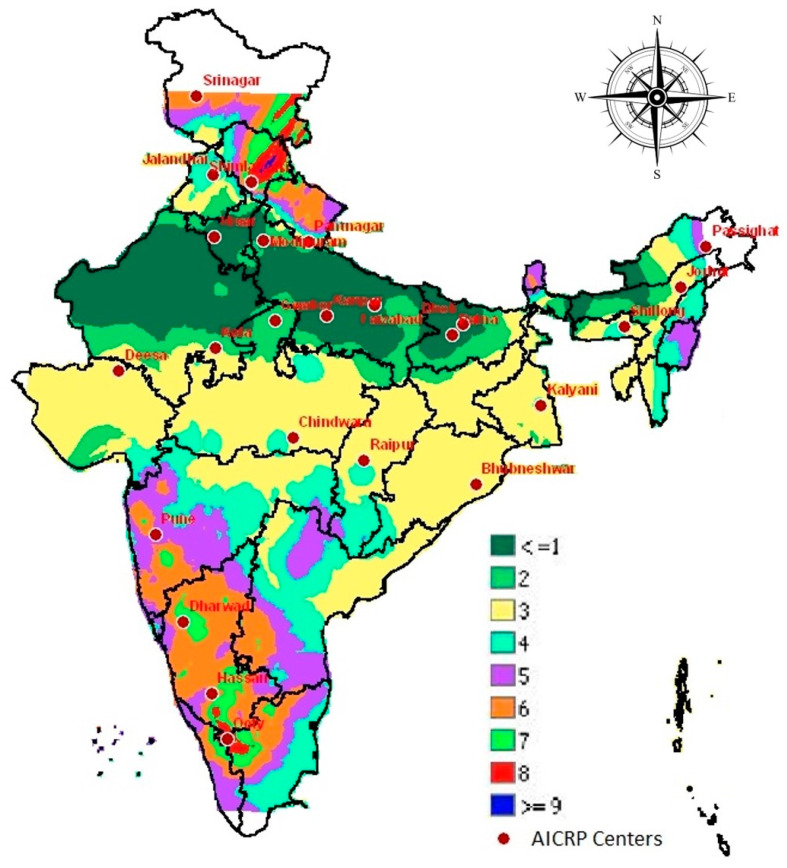
Thematic map representing the spatial distribution of different classes of potato growing regions of India.

The thematic map of the interpolated surface of the classes shows that in the plains, the homogenous classes are spread out across longitudes, i.e., most of the area in Rajasthan, Haryana, Uttar Pradesh and Bihar falls between latitudes 26° N to 30° N is in the class 1 and in the fringe areas of class 1 is in class 2. Most parts of central India covering Gujarat, Madhya Pradesh, Chhattisgarh, Odisha, Jharkhand and West Bengal between latitudes 23° N to 26° N are in class 3. In the plateau and hills, the latitudinal spread of the class is not seen and different classes are interspersed together due to the altitudinal gradient (Figure 4).

## 4. Discussion

The performance of a genotype is a function of its genetic makeup and that of the environment in which it is grown. Therefore, a lot of emphasis has been given to multi-location testing of advanced genetic materials to study their interaction and determine their adaptation domains. However, the emphasis of breeders hitherto to base their conclusion on the performance against check varieties in a given location are reliable only within the environments tested, and this may not be the case when genotypes are to be recommended for large TPE [20]. However, in recent years, the availability of meteorological data at high resolutions as well as developments in the field of crop modeling and GIS have prompted scientists to use these tools for better targeting of genotypes to different environments. Li et al. [21] used a crop model and a limited number of multi-environment trials to study the performance and stability of Green Super Rice. Though the use of crop models for virtual multi-environmental trials is very promising, it is possible only in cases where detailed physiological data of the genotype is available. However, this is not the case when advanced hybrids are being tested in multi-locations, since a large number of hybrids and the limited availability of seed material limit the possibility of conducting detailed physiological studies to determine the genotypic coefficients. In such situations, delineating zones based on climate similarity would be more appropriate. Teixeira et al. [22] characterized the wine grape growing regions based on thermohydrological conditions. Tonietto and Carbonneau [14] and Conceicao and Tonietto [23] developed a multicriteria climate-based classification system for grape growing worldwide. The classification into similar environments of maize testing sites has been carried out earlier [1,24] and in barley by Beillouin et al. [25]. Hyman et al. [8] have used spatial analysis to support the geographic targeting of genotypes to different environments. These studies emphasize the need to use climatic and edaphic factors for better targeting of genotypes to the environments using modern IT tools like GIS and crop models. In this study, we used the climatic data to delineate similar zones because climatic factors are the primary determinant determining the suitability of a location for growing a particular crop/variety of potato which is grown largely under irrigated conditions but in diverse soil types in India.

With regard to the choice of meteorological variables, the first determinant for potato cultivation in the sub-tropics is temperature. It not only determines whether potato can be grown at all, and if it can be, it determines its duration and productivity. The optimum mean temperature for potato has been reported to be 18 °C [26] while its response to temperature was found to be hyperbolic with an optimum at about 18 °C [27]. Therefore, the mean temperature was chosen as one of the meteorological variables. Apart from mean temperature, mean night temperature is also very important for potato, especially in the subtropics [28]. Furthermore, two temperature-derived variables including growing degree days (GDD) and P days have also been used to categorize the potato growing season. The GDD has been included because it indicates the total length of the growing period, while P days represent the effective length of the growing period. The GDD or the thermal time is most often used to account for crop phenology. Worthington and Huchinson [29] developed a growing degree day calendar for potato production in northeast Florida. GDD-based recommendations have been shown to give an accurate prediction of planting dates and yield in many crops such as pea [30], broccoli [31], and cucumber [32]. This index has also been used in the INFOCROP-Potato crop model to compute the development stages. Therefore, in this study the total GDD was also used as an index of the available growing period. One of the criticisms of the GDD concept is that it considers the relationship between temperature and growth to be linear, while in actuality this is not the case. Therefore, Sands (1979) developed the P days concept to account for the non-linear relationship between temperature and growth with the maximum response being at the optimum and decreasing towards both the lower and higher extremes. Connell et al. [33] used this concept to model the development of LAI based on P days, while Sands et al. [18] modeled the yield of potato based on P days. The P days concept has also been used to model the growth of many pests and diseases [34,35].

In addition to the temperature-derived variables, the mean solar radiation of the growing season was also included since it is a major determinant of the potential productivity of the season. Almost all radiation-based growth engines of crop models determine the total dry matter production based on the conversion of total intercepted radiation using a conversion efficiency [36]. Since potato in India is grown in widely varying environments, the altitudinal differences between the locations also determine to a large extent the climatic characteristics of the growing season and thus on the growth and development. Therefore, the altitude was included as one of the variables since it represents the growing season which shifts from summer in the hills to winter in the plains. The low within-class and high between-classes variance obtained in the study shows that the AHC clustering technique using these six parameters adequately accounted for the climatic variability of the locations, and by using these parameters we can efficiently classify the locations into similar zones (Table 2).

The study has revealed that the methodology of determining the plausible potato growing period and determining their climate similarity using the clustering technique can effectively delineate the TPE for potato genotypes performing well in different testing environments in India. It has also established that the six parameters (Altitude, Physiological days, Growing degree days, Mean night temperature, Mean total temperature and Mean radiation) used in the study categorized the potato growing regions efficiently under Indian conditions. The study also shows that class no. 3 represented the largest potato growing area, followed by class no. 1. However, class no. 7 also represented a sizeable area, and this area has the highest mean night and mean total temperatures. In the sub-tropical climate of tropical and sub-tropical regions, it is the mean night temperature which is affects tuberization. Under climate change scenarios, potato cultivation will be most affected in such areas, having night temperatures >20 °C [37,38,39], and therefore potato breeding in this subcontinent has to prioritize class 7 followed by class no. 2 in future breeding strategies. Thus, to further expand the potato acreage in India, the emphasis should be given to extending the areas classified under classes 7 and 2.

## Figures and Tables

**Table 1:** Summary of class-wise mean and range values of meteorological variables of 1253 potato growing locations in India.

Class	Number of Locations	Meteorological Variables					
		Altitude (m)	Physiological Days	Growing Degree Days	Mean Night Temperature (°C)	Mean Total Temperature (°C)	Mean Radiation (KJ/day/m^2^)
1	399	211.2 (2.0–1359.0)	849.0 (469.8–1065.2)	1749.0 (1124.0–2637.9)	15.5 (12.7–22.7)	19.2 (16.3–26.0)	18,684.1 (17,136.7–19,424.2)
2	45	567.2 (4.0–1729.0)	813.6 (467.0–1042.3)	1641.4 (1025.1–2300.6)	15.9 (12.5–21.4)	18.6 (15.8–23.5)	16,720.4 (15,471.8–17,775.0)
3	540	279.2 (4.0–1407.0)	798.3 (355.2–1050.4)	1856.6 (939.2–2383.8)	17.8 (12.8–24.3)	21.6 (16.9–25.1)	20,287.6 (19,184.8–21,441.1)
4	23	374.3 (182.0–900.0)	671.3 (488.5–928.7)	1546.8 (1282.7–1781.1)	17.8 (14.7–21.2)	21.0 (14.3–23.2)	14,755.5 (13,845.3–15,501.4)
5	17	1036.3 (181.0–2311.0)	1046.1 (748.2–1127.0)	1806.8 (1316.2–1999.4)	17.9 (13.3–19.3)	18.9 (11.0–20.7)	7594.6 (6539.2–8346.7)
6	12	1125.7 (282.0–2145.0)	940.3 (549.7–1121.2)	1718.5 (1123.1–2091.2)	17.0 (13.3–20.0)	19.9 (14.92–21.46)	9520.4 (8947.5–10,221.7)
7	166	517.5 (6.0–1100.0)	704.6 (352.9–1027.5)	1802.3 (984.4–2368.6)	19.8 (14.4–22.4)	23.3 (17.5–25.9)	21,832.8 (10,929.2–23,222.8)
8	15	315.6 (9.0–782.0)	841.6 (410.0–1090.9)	1748.6 (823.2–2151.2)	18.0 (14.7–21.4)	19.6 (11.7–23.0)	12,912.4 (12,018.6–13,683.1)
9	16	1207.1 (247.0–1928.0)	929.3 (718.9–1108.6)	1711.9 (1190.0–2142.6)	16.6 (11.3–20.8)	17.8 (10.8–22.5)	11,257.6 (10,301.7–12,533.3)
10	7	1535.1 (1183.0–1821.0)	756.8 (545.6–971.0)	1404.7 (1036.4–1594.8)	14.8 (12.0–18.1)	17.6 (15.3–21.8)	13,709.4 (12,997.5–14,179.16)
11	3	1946.0 (1689.0–2127.0)	907.4 (853.4–941.4)	1699.4 (1489.7–1918.0)	16.1 (15.2–17.3)	18.5 (17.5–20.0)	15,189.0 (14,908.3–15,527.9)
12	1	1959.0	836.6	1341.8	12.7	15.2	22,594.2
13	5	605.8 (233.0–790.0)	709.2 (505.3–1116.5)	1715.5 (1482.4–2353.8)	18.8 (13.2–20.4)	22.8 (17.9–24.3)	24,443.3 (24,055.1–24,968.0)
14	2	2158.0 (2079.0–2237.0)	895.0 (871.7–918.3)	1485.9 (1426.9–1544.9)	13.7 (13.3–14.2)	16.4 (15.9–16.9)	19,208.8 (19,005.0–19,412.5)
15	2	1962.5 (1941.0–1984.0)	895.3 (851.6–938.9)	1736.6 (1487.3–1986.6)	15.7 (13.9–17.5)	9.4 (7.3–11.4)	5652.1 (5084.4–6219.9)
Max		2311.0	1127.0	2637.9	24.3	26.0	24,968.0
Min		2.0	352.9	823.2	11.3	7.3	5084.4
Average		351. 3	807.2	1792.8	17.2	20.8	19,226.0
Standard deviation		343.1	155.5	306.1	2.2	2.2	2649.5

**Table 2:** Class-wise clustering of AICRP (Potato) centres and the number of potato growing locations they represent.

Class No.	Number of AICRP (P) Centres in the Specific Class	All India Coordinated Research Project (Potato)	
		Name of the AICRP (P) Centres ^#^	Number of Locations in the Class
1	7	Patna (25.59°; 85.13°), Meerut (28.98°; 77.70°), Faizabad (26.77°, 82.14°), Hassan (13.01°; 76.10°), Hisar (29.15°; 75.72°), Kanpur (26.45°; 80.33°) and Kota (25.21°; 75.86°)	399
2	-	-	45
3	4	Bhubaneshwar (20.29°; 85.82°), Deesa (24.26°; 72.19°), Gwalior (26.22°; 78.18°) and Raipur (21.25°; 81.62°)	540
4	-	Shillong (25.57°; 91.89°)	23
5	1	Shimla (31.10°; 77.17°)	17
6	1	-	12
7	2	Dharwar (15.46°; 75.01°) and Pune (18.52°; 73.85°)	166
8	4	Jorhat (26.75°; 94.20°), Kalyani (22.98°; 88.43°), Pantnagar (29.02°; 79.49°) and Pasighat (28.06°; 95.32°)	15
9	-	-	16
10	1	Srinagar (J & K) (34.08°; 74.80°)	7
11	1	Ranichauri/Pauri (30.14°; 78.77°)	3
12	-	-	1
13	1	Jalandhar (31.33°; 75.58°)	5
14	1	Ootacamund (11.40°; 76.69°)	2
15	-	-	2
Total			1253

**^#^** Values in the parentheses indicate the latitude & longitude of the specific AICRP (P) centre.

## Data Availability

Raw data of this research study are available by contacting the authors.

## Supplementary Materials

The following supporting information can be downloaded at: https://www.mdpi.com/article/10.3390/life12101619/s1, Table S1: Details of 15 Classes classified based on meteorological variables of 1253 potato growing locations in the country.
