# Supplementary material for: Characterizing the Potato Growing Regions in India Using Meteorological Parameters
Source: Life (Basel). 2022 Oct 17;12(10):1619. doi: 10.3390/life12101619 (PMC9605082; doi:10.3390/life12101619)
Supplement: Supplementary file 1 [file life-12-01619-s001.zip › life-1921273-supplementary.pdf]

Suppl. File:
Details of 15 Classes classified based on meteorological variables of 1253 potato growing locations in the country.

Class #1(Locations: 386)

Sr No	Station	Latitude	Longitude	Altitude	P days (7,21,30)	GDD (4 deg C base temp)	Mean Night temp	Mean T	Mean Radiation (Kj/day/m2)
1	Aliganj	26.90	80.94	108	600.35	1221.55	14.79	18.95	18556.10
2	Alwar	27.55	76.63	261	898.05	1888.40	15.97	19.77	18756.67
3	Ambah	26.70	78.22	166	875.47	1855.90	15.47	19.50	19036.67
4	Amber	25.20	85.52	46	853.65	1749.05	14.76	18.61	18273.33
5	Amethi	26.15	81.81	93	854.71	1804.15	14.86	19.07	18906.67
6	Antah	25.15	76.31	243	916.50	1983.80	20.32	20.57	19090.83
7	Anupshahr	28.35	78.26	196	710.84	1416.50	14.28	18.21	18070.00
8	Aonla	28.26	79.16	167	919.49	1774.95	15.57	18.82	18285.00
9	Arrah	25.56	84.66	53	932.49	1823.10	15.67	19.23	19168.33
10	Atrauli	28.03	78.29	189	708.74	1400.20	14.23	18.18	18108.08
11	Auraiya	26.46	79.51	137	677.25	1356.75	14.84	18.79	18384.78
12	Ayodhya	26.79	82.20	94	900.21	1775.15	14.78	18.83	19050.83
13	Azamgarh	26.07	83.19	74	878.19	1802.85	14.85	19.06	19223.33
14	Badausa	25.21	80.60	114	879.27	1858.60	15.44	19.52	19308.33
15	Badli	28.57	76.80	221	712.78	1399.30	14.23	18.03	17968.00
16	Bagdogra	26.69	88.31	133	890.59	1955.85	16.59	20.33	19150.83
17	Bagha	24.19	88.83	8	892.34	1869.95	15.65	19.62	18733.33
18	Baghpat	28.94	77.22	217	621.96	1237.80	15.36	19.14	18093.90
19	Baharaich	27.57	81.59	111	860.42	1895.95	15.53	19.83	18329.17
20	Baheri	28.77	79.49	181	921.47	1767.35	15.60	18.76	18216.67
21	Bairagnia	26.73	85.27	62	880.36	1753.00	14.63	18.64	18985.00
22	Bairat	27.43	76.18	433	904.77	1892.20	16.16	19.80	18530.00
23	Bakhtiarpur	25.72	86.59	34	888.07	1684.35	14.30	18.07	19157.50
24	Bali	25.19	73.28	296	895.62	2005.95	16.71	20.75	19338.33
25	Balichak	22.36	87.55	16	876.48	2201.70	18.73	19.32	17683.45
26	Balipara	26.82	92.77	70	947.00	1855.40	16.24	19.50	18998.33
27	Ballabhgarh	28.34	77.32	198	712.14	1404.35	14.25	18.08	18071.00
28	Ballia	25.75	84.14	58	927.02	1851.65	15.79	19.46	18893.33
29	Balotra	25.83	72.24	113	892.89	2157.85	17.96	22.02	19135.83
30	Banda	25.28	80.22	25	922.71	1833.50	15.60	19.31	19205.83

31	Bansdih	25.88	84.21	56	922.71	1833.50	15.60	19.31	19205.83
32	Bansi	27.15	82.93	85	698.42	1354.85	14.51	18.30	18620.00
33	Baran	25.10	76.51	270	909.42	1871.25	15.67	19.63	19226.67
34	Bareilly	28.36	79.43	168	922.18	1772.15	15.58	18.80	18260.83
35	Bareilly Sadar	28.31	79.44	161	920.57	1763.20	15.51	18.73	18245.83
36	Barh	25.47	85.70	34	865.63	1632.70	14.17	17.64	19123.33
37	Barmer	25.75	71.41	167	858.89	1934.55	15.87	20.15	19228.33
38	Baroda	22.30	73.18	43	888.83	1837.25	15.63	19.34	18557.50
39	Barwasagar	25.37	78.74	224	840.64	1860.00	15.12	19.53	19259.17
40	Basanti	22.20	88.71	6	883.93	1752.25	15.03	18.64	18710.83
41	Baseri	26.74	77.53	200	845.21	1757.25	14.58	18.68	18821.67
42	Basohli	32.50	75.81	552	599.96	1311.05	16.94	20.04	18893.90
43	Basti	26.81	82.76	78	867.88	1803.85	14.91	19.07	18753.33
44	Baswa	27.14	76.58	287	868.53	1863.65	15.43	19.56	18570.00
45	Beawar	26.10	74.31	451	895.66	1862.90	15.76	19.56	18853.33
46	Beri	28.70	76.58	223	840.36	1806.75	15.06	19.09	18460.83
47	Bhadra	29.10	75.10	201	816.71	1911.10	15.62	19.96	18250.83
48	Bhainsdehi	21.64	77.63	760	971.28	2097.45	18.48	21.51	18985.00
49	Bhainswal	29.44	77.00	230	889.18	1676.40	14.61	18.00	17974.17
50	Bharatpur	27.21	77.48	180	885.64	2039.40	17.63	21.32	19083.05
51	Bharthana	26.75	79.21	148	837.77	1857.80	15.44	19.52	19184.17
52	Bhilwara	25.32	74.58	424	875.44	1853.30	15.25	19.48	19352.50
53	Bhivandi	19.25	73.09	9	488.89	1438.70	20.91	24.61	21782.86
54	Bhognipur	26.20	79.81	121	678.24	1386.00	15.18	19.11	18816.30
55	Biana	29.83	77.11	251	860.69	1812.05	15.10	19.13	18786.67
56	Bidhuna	26.80	79.50	138	841.39	1724.75	14.09	18.41	18971.67
57	Bilgram	27.11	80.02	133	734.79	1405.60	14.27	18.10	18674.00
58	Bilhaur	26.84	80.06	119	839.44	1682.90	13.79	18.06	18970.00
59	Bisauli	28.29	78.93	172	860.68	1673.90	14.07	17.98	18107.50
60	Biswan	27.49	80.99	126	674.46	1435.80	14.50	18.40	18630.00
61	Budaun	28.03	79.12	163	903.71	1750.55	14.83	18.62	18248.33
62	Budhana	29.28	77.47	231	890.12	1777.70	15.43	18.85	17838.33
63	Bulandshahr	28.40	77.84	198	855.11	1766.10	15.05	18.75	18160.83
64	Bundi	25.43	75.64	267	903.28	1988.85	16.82	20.61	19204.17
65	Chamarajnagar	11.93	76.94	695	1008.28	2091.55	18.75	21.46	19250.83
66	Chandor	15.26	74.04	2	977.66	2045.60	18.14	21.08	19150.83
67	Chapra	25.78	84.75	49	930.75	1931.85	16.59	20.13	19180.83

68	Charduar	26.52	92.47	87	945.72	1850.50	16.20	19.45	18970.00
69	Chhibramau	27.15	79.50	138	873.40	1815.65	15.43	19.16	18486.67
70	Chhotakosi	27.47	77.26	177	887.57	1763.95	15.14	18.73	18672.50
71	Chickballapur	13.43	77.73	920	990.64	2231.75	20.27	22.63	19060.83
72	Chinatapalli	17.22	78.61	512	1021.32	1757.20	16.56	18.68	18440.83
73	Chirawa	28.24	75.65	312	694.26	1573.55	15.50	19.78	18538.00
74	Chomu	27.17	75.72	486	892.97	1827.30	15.56	19.26	18582.50
75	Chunar	25.10	82.87	9	863.81	1777.90	14.73	18.85	19184.17
76	Churu	28.29	74.96	282	695.00	1457.45	15.28	19.22	18701.04
77	Dalmau	26.07	81.06	105	892.53	1840.50	15.36	19.37	19210.83
78	Darbhanga	26.11	85.90	34	902.64	1893.60	16.03	19.81	18932.50
79	Dataganj	28.03	79.41	149	907.23	1761.65	14.97	18.71	18236.67
80	Dausa	26.89	76.34	345	874.71	1829.85	15.17	19.28	18864.17
81	Dayanathpur	28.71	77.93	202	710.45	1403.65	14.22	18.08	18108.00
82	Dehri	20.15	72.74	5	911.59	1891.65	16.13	19.80	19397.50
83	Delhi Sadar	28.66	77.21	238	706.26	1365.00	14.01	17.83	17962.63
84	Deoband	29.69	77.68	253	876.37	1766.65	15.12	18.76	17722.50
85	Deogaon	25.74	82.99	72	871.87	1757.65	14.59	18.68	19265.00
86	Deoria	26.49	83.78	67	774.58	1459.80	14.16	17.81	19077.36
87	Derapur	26.49	83.78	67	675.94	1366.15	14.95	18.89	18788.04
88	Desuri	25.28	73.57	384	894.32	1941.20	16.31	20.21	19367.50
89	Dhamdaha	25.74	87.18	36	900.40	1904.65	16.08	19.91	19095.00
90	Dhampuir	29.31	78.51	235	823.67	1575.25	13.16	17.16	17745.00
91	Dhandaus	27.06	79.17	151	602.09	1228.00	14.86	19.02	18626.83
92	Dholpur	26.70	77.89	184	855.72	1817.55	15.12	19.18	18860.00
93	Dhubri	26.02	89.97	22	915.65	1879.55	16.15	19.70	18722.50
94	Didwana	27.40	74.58	352	581.07	1184.00	14.05	18.49	18426.83
95	Digri	26.56	70.94	253	813.36	1719.50	15.95	19.67	19297.27
96	Doomni	26.72	91.31	66	826.29	1593.85	16.32	19.36	17596.15
97	Dubari	26.21	83.73	58	826.29	1593.85	16.32	19.36	17596.15
98	Etah	27.56	78.66	165	872.00	1707.25	14.46	18.26	18746.67
99	Etawah	26.81	79.00	135	859.51	1825.45	15.20	19.25	18795.83
100	Faizabad	26.77	82.14	96	881.72	1763.45	14.81	18.73	18904.17
101	Falakata	26.52	89.20	48	905.29	1899.95	16.17	19.87	18881.67
102	Faridpur	28.21	79.54	164	919.81	1764.60	15.51	18.74	18285.83
103	Farrukhabad	27.38	79.59	145	838.02	1731.85	14.20	18.47	18656.67
104	Farrukhanagar	28.45	76.82	220	842.69	1677.95	13.99	18.02	18080.83

105	Fatehabad	29.51	75.45	202	821.30	1891.15	15.52	19.79	18149.17
106	Fatehgarh	27.37	79.62	138	853.29	1857.95	15.21	19.52	18765.00
107	Fatehgarha	27.22	79.38	156	841.20	1734.00	14.24	18.48	18651.67
108	Fatehpur	25.92	80.81	112	882.67	1720.15	14.31	18.37	18760.00
109	Fatehpur Sikri	27.09	77.67	180	882.12	1768.85	15.10	18.77	18806.67
110	Fatehpura	24.61	73.69	576	907.31	1863.95	15.70	19.57	18969.17
111	Firozabad	27.16	78.40	160	878.31	1702.55	14.58	18.22	18787.50
112	Firozpur	30.93	74.62	188	863.46	1861.75	15.35	19.55	18603.33
113	Gangapur	19.70	75.01	477	874.83	1771.10	14.71	18.79	19359.17
114	Gangapura	27.96	81.78	118	888.38	1870.60	15.61	19.62	19036.67
115	Gangapuraa	25.13	74.15	496	869.56	1837.05	15.13	19.34	19372.50
116	Gangarampur	25.40	88.53	24	944.14	1981.85	16.92	20.55	19307.50
117	Gauhati	26.14	91.74	43	944.54	1876.55	16.21	19.67	19286.67
118	Ghatampur	26.15	80.17	124	885.61	1838.60	15.43	19.36	19066.67
119	Ghaziabad	28.67	77.45	216	862.13	1685.35	14.21	18.08	17905.00
120	Ghazipur	25.58	83.58	64	881.11	1800.85	15.17	19.04	19149.17
121	Ghosi	26.12	83.54	64	879.27	1812.35	14.93	19.14	19211.67
122	Girwan	25.31	80.39	162	871.50	1825.00	15.15	19.24	19325.00
123	Goalpara	26.16	90.63	43	962.00	1960.50	17.02	20.37	19081.67
124	Gohana	29.14	76.69	226	874.51	1948.95	16.52	20.27	17980.00
125	Golaghat	26.52	93.96	82	961.28	1827.25	15.97	19.26	18775.00
126	Gonda	27.13	81.96	109	680.37	1820.60	17.91	22.07	18007.92
127	Gopalpura	26.88	75.78	407	894.26	1736.20	14.99	18.50	18357.50
128	Gorakhpur	26.76	83.37	70	883.08	1850.60	15.42	19.46	18765.00
129	Gunnaur	28.24	78.44	172	710.70	1398.10	14.26	18.16	18057.58
130	Gurgaon	28.46	77.03	226	710.41	1376.60	14.01	17.81	18010.00
131	Hamirpura	22.07	74.09	226	846.09	1858.95	15.17	19.52	19180.83
132	Handia	25.36	82.19	86	883.91	1749.45	14.60	18.61	19215.00
133	Hansi	29.10	75.96	210	819.63	1916.95	15.71	20.01	18264.17
134	Hanumangarh	29.61	74.29	174	577.11	1334.40	16.56	20.52	18460.49
135	Hapur	28.73	77.78	214	861.22	1683.35	14.18	18.06	17900.00
136	Haraiya	26.07	83.17	58	911.41	1820.80	15.24	19.21	19023.33
137	Hardoi	27.40	80.13	141	698.16	1749.40	16.91	21.19	18382.35
138	Hasanganj	26.87	80.94	110	886.21	1825.90	15.32	19.25	18914.17
139	Hasanpur	28.72	78.28	205	886.21	1825.90	15.32	19.25	18914.17
140	Hasanpura	26.92	75.77	426	884.74	1748.50	14.60	18.60	19270.00
141	Hassan	13.01	76.10	944	739.28	1736.75	21.21	23.34	19114.44

142	Hassanpur	29.06	77.07	222	890.70	1814.35	15.55	19.15	18345.83
143	Hata	26.74	83.74	73	888.86	1679.65	14.17	18.03	19039.17
144	Hathras	27.61	78.05	183	884.60	1751.05	15.00	18.63	18699.17
145	Hattin	28.04	77.23	189	710.45	1396.85	14.15	18.01	18117.00
146	Hindaun	26.75	77.03	227	898.32	1874.05	15.80	19.65	19135.83
147	Hindoli	25.57	75.50	294	920.64	2052.35	17.74	21.14	19044.17
148	Hissar	29.15	75.72	203	822.16	1913.75	15.70	19.98	18242.50
149	Iglas	27.71	77.94	178	887.24	1752.65	15.05	18.64	18686.67
150	Indaagarh	25.43	76.12	248	906.19	1871.45	15.67	19.63	18994.17
151	Isarda	26.17	76.03	276	894.14	1919.55	16.09	20.03	19254.17
152	Itawah	26.81	79.00	135	904.45	1895.00	15.83	19.83	19045.00
153	Jagadhari	30.17	77.30	283	871.78	1698.75	14.62	18.19	17550.83
154	Jaipura	30.78	76.03	251	853.44	1911.00	16.06	19.96	19334.17
155	Jaisalmer	26.92	70.91	237	872.61	1941.55	16.33	20.21	18838.33
156	Jaitram	26.12	73.57	297	879.02	1872.80	15.29	19.64	18990.83
157	Jalalabad	27.72	79.66	138	875.09	1749.10	14.44	18.61	18647.50
158	Jalalpur	25.86	79.81	119	925.95	1796.90	15.60	19.01	18786.67
159	Jalaun	26.15	79.33	137	674.44	1376.35	15.02	19.00	18870.65
160	Jalesar	27.47	78.31	164	870.57	1699.30	14.40	18.19	18750.00
161	Jalpaiguri	26.54	88.72	75	910.17	1954.90	16.62	20.32	18825.00
162	Jamwa Ramgarh	27.02	76.00	407	592.90	1129.80	13.78	17.83	18580.49
163	Jarauda	28.89	78.01	215	887.08	1729.15	15.03	18.44	17749.17
164	Jatusana	28.33	76.54	236	721.50	1511.90	15.81	19.47	18736.73
165	Jaunpur	25.75	82.70	75	880.76	1742.20	14.54	18.55	19049.17
166	Jawaja	26.07	74.31	454	894.49	1768.05	14.99	18.77	19231.67
167	Jhunjhunu	28.13	75.40	331	587.34	1219.10	14.49	18.92	18092.68
168	Jiwanpur	30.41	73.95	170	878.00	1802.75	14.85	19.06	19205.00
169	Jodhpur	26.24	73.02	213	870.71	2044.35	17.07	21.07	18979.17
170	Jowai	25.45	92.21	1359	894.03	2051.55	18.67	22.03	19123.68
171	Jowari	25.26	92.12	139	894.03	2051.55	18.67	22.03	19123.68
172	Kachugaon	26.54	93.98	88	928.47	1940.95	16.79	20.21	19086.67
173	Kaimganj	27.55	79.33	147	604.61	1229.30	14.90	19.04	18509.76
174	Kairana	29.39	77.20	233	899.39	1774.80	15.43	18.82	17815.00
175	Kaithal	29.79	76.40	234	831.18	1828.60	15.14	19.27	18060.00
176	Kalchini	26.69	89.45	107	886.92	1933.65	17.06	20.56	18564.96
177	Kalpi	26.12	79.75	108	679.88	1384.30	15.19	19.09	18821.74

178	Kamasin	25.51	80.91	100	913.08	1904.80	16.05	19.91	19060.00
179	Kamen	27.39	77.16	183	887.57	1821.70	15.59	19.21	18401.67
180	Kannauj	27.05	79.91	124	832.51	1641.25	13.59	17.71	18486.67
181	Kanpur	26.45	80.33	128	837.05	1646.80	13.60	17.76	19008.33
182	karauli	26.49	77.02	275	821.57	1685.55	13.72	18.08	19098.33
183	Karchana	25.28	81.95	88	873.16	1835.30	15.24	19.33	19096.67
184	Kasganj	27.81	78.65	173	614.17	1249.10	15.60	19.47	18701.23
185	Kashipur	29.21	78.96	232	908.03	1810.30	15.74	19.12	17872.50
186	Kekri	25.97	75.15	355	907.64	1844.30	15.51	19.40	19025.83
187	Kerakat	25.64	82.92	73	872.77	1760.30	14.61	18.70	19288.33
188	Khaga	25.77	81.10	102	881.12	1733.60	14.48	18.48	19038.33
189	Khair	27.94	77.84	186	872.62	1686.25	14.37	18.09	18668.33
190	Khairagarha	26.56	77.48	192	853.12	1787.05	14.87	18.93	18803.33
191	Khajwa	24.92	79.93	200	880.47	1781.70	14.70	18.88	19196.67
192	Khalilabad	26.78	83.07	71	901.28	1735.15	14.70	18.49	18878.33
193	Khamera	23.79	74.46	233	956.45	2098.85	18.50	21.52	18630.00
194	Khandar	27.21	77.98	171	866.81	1788.40	14.71	18.94	19250.00
195	Khatima	28.92	79.97	207	893.85	1688.10	14.59	18.10	17945.83
196	Kheri	28.17	80.63	137	853.92	1733.55	14.48	18.48	18250.00
197	Khetri	28.00	75.79	393	571.49	1123.95	13.36	17.76	18126.83
198	Khol	28.20	76.40	288	800.81	1611.80	14.85	18.69	18837.27
199	Khutar	28.21	80.27	157	877.49	1697.00	14.55	18.18	18215.83
200	Kishangarh	26.59	74.86	452	878.99	1936.85	16.32	20.17	18485.00
201	Kotah	25.21	75.86	259	910.41	1998.35	16.97	20.69	19237.50
202	Kotkasim	28.03	76.72	259	888.45	1832.40	15.68	19.30	18245.83
203	Kotputli	27.70	76.20	363	899.29	1872.65	15.97	19.64	18737.50
204	Kuakhera	29.66	77.38	248	879.08	1643.35	14.33	17.73	18012.50
205	Kumargram	26.61	89.83	65	909.42	1850.00	15.59	19.45	18916.67
206	Kunch	26.00	79.16	146	668.61	1347.60	14.62	18.69	19064.13
207	Kunda	25.72	81.52	94	887.13	1731.35	14.54	18.46	19029.17
208	Kundhra	28.42	79.40	184	923.11	1766.10	15.56	18.75	18165.83
209	Kurtha	25.13	84.81	69	923.70	1864.65	15.91	19.57	19069.17
210	Lalsot	26.56	76.33	310	875.05	1743.25	14.52	18.56	19030.00
211	Lathi	21.73	71.39	122	812.57	1474.30	12.72	16.32	18244.17
212	Lucknow	26.85	80.95	112	888.21	1787.80	14.79	18.93	19023.33
213	Lumding	25.75	93.17	125	899.15	1920.35	16.46	20.04	19348.33
214	Lunkaransar	28.50	73.75	193	617.27	1371.65	15.13	19.46	18084.27

215	Machhlishahr	25.69	82.41	85	867.58	1759.95	14.58	18.70	19259.17
216	Madhwapur	25.44	81.86	94	872.77	1798.95	15.18	19.02	19015.00
217	Magarwara	26.50	80.43	118	837.04	1838.30	14.92	19.35	19267.50
218	Maharajganj	27.14	83.56	83	865.28	1789.50	14.80	18.95	19040.83
219	Mahua	25.81	81.39	36	913.06	1903.15	16.36	19.89	19287.50
220	Mahul	19.03	72.89	10	873.71	1769.30	14.69	18.78	19021.67
221	Mahwa	27.03	76.92	225	900.76	1950.60	16.59	20.29	18938.33
222	Maibong	25.26	93.14	505	863.41	1718.15	14.79	18.47	18700.84
223	Mainpuri	27.22	79.02	224	838.29	1624.90	13.48	17.57	18300.00
224	Malpura	26.30	75.36	326	876.73	1841.75	15.29	19.38	18885.00
225	Manatu	24.23	84.40	331	838.71	1949.10	16.16	20.28	18792.50
226	Mandawar	27.15	76.85	234	879.15	1941.70	16.35	20.21	18475.83
227	Mangrol	25.33	76.51	226	903.86	1942.30	16.15	20.22	19424.17
228	Manjhanpur	25.52	81.37	105	908.70	1718.10	14.90	18.35	19187.50
229	Mankapur	27.03	88.21	92	896.82	1780.05	14.86	18.87	18737.50
230	Marhaura	25.96	84.86	47	880.58	1653.55	14.00	17.81	19138.33
231	Mariahu	25.60	82.58	70	867.26	1748.30	14.48	18.60	19315.83
232	Mathabhango	26.34	89.21	52	944.04	1921.35	16.35	20.04	18905.00
233	Mathura	27.49	77.67	180	886.72	1752.65	15.03	18.64	18731.67
234	Mauganj	24.66	81.88	345	676.14	1780.00	17.49	21.84	18889.00
235	Mauhaha	25.41	80.07	200	831.16	1751.60	14.23	18.63	19325.83
236	Mawana	29.09	77.92	225	876.45	1696.05	14.21	18.17	17840.83
237	Meerut	28.98	77.7	225	873.84	1648.85	14.21	17.77	18085.83
238	Meja	25.14	81.98	86	864.29	1712.35	14.30	18.30	19313.33
239	Mekliganj	26.34	88.91	57	919.17	1838.90	15.68	19.36	19085.00
240	Mertacity	26.64	74.03	312	892.72	1887.95	15.92	19.77	19078.33
241	Mirzapur	25.12	82.56	79	863.96	1779.60	14.74	18.86	19174.17
242	Misrikh	27.42	80.52	138	866.07	1740.00	14.49	18.53	18459.17
243	Mohanlalganj	26.68	80.98	102	859.59	1760.20	14.53	18.70	18967.50
244	Moradabad	28.83	78.77	193	872.09	1735.75	14.94	18.50	18148.33
245	Moth	25.72	78.95	169	872.59	1934.05	15.99	20.15	19324.17
246	Motihari	26.64	84.9	58	863.83	1727.70	14.24	18.43	19048.33
247	Moyna	22.27	87.76	5	647.04	2637.90	22.73	26.02	19283.33
248	Mozamabad	26.67	75.36	334	869.24	1892.35	15.79	19.80	19005.83
249	Muhamdi	27.95	80.2	150	889.55	1762.25	14.95	18.72	18355.83
250	Mungaoli	24.41	78.09	408	901.45	1859.65	15.58	18.03	18417.69
251	Musafirkhana	26.37	81.8	97	684.02	1349.35	14.94	18.71	18706.52

252	Nagore	27.19	73.74	304	793.36	1752.70	16.23	20.12	19247.71
253	Najafgarh	28.6	76.98	217	874.25	1709.30	14.60	18.28	18349.17
254	Najibabad	29.61	78.34	264	892.66	1678.50	14.45	18.02	17725.83
255	Nangloi	28.68	77.06	218	712.09	1392.80	14.18	17.97	17984.00
256	Nanpara	27.86	81.49	123	907.00	1771.95	14.93	18.80	18507.50
257	Naraina	28.63	77.13	224	841.56	1743.95	14.36	18.57	18975.00
258	Narela	28.85	77.09	223	711.21	1393.45	14.17	17.97	17943.00
259	Nawa	27.02	75	384	605.14	1303.10	14.68	18.36	18289.01
260	Nawabganj	28.54	79.63	172	919.54	1760.90	15.48	18.71	18210.00
261	Nawanshahr	31.09	76.03	239	746.92	1678.55	16.04	19.72	17758.88
262	Neemkathana	27.73	75.78	448	872.18	1828.65	15.31	19.27	18430.83
263	New Delhi	28.61	77.2	205	873.42	1673.00	14.43	17.98	18383.33
264	Nighasan	28.23	80.87	135	884.28	1676.50	14.18	18.00	18107.50
265	Niwari	28.87	77.53	218	887.04	1859.80	15.53	19.53	18935.00
266	Nohar	29.17	74.76	183	820.63	1806.50	14.86	19.09	17890.83
267	Nokh	27.55	73.47	320	851.95	1849.05	15.29	19.44	18511.67
268	Nurpur	32.3	75.88	572	590.04	1221.95	15.74	18.95	18819.51
269	Orai	26	79.44	127	847.13	1806.75	14.87	19.09	19334.17
270	Pachpadra	25.92	72.25	106	758.39	1890.65	18.00	22.22	19061.54
271	Padampur	29.7	73.62	164	578.11	1319.45	16.44	20.34	18407.41
272	Padrauna	26.89	83.97	75	850.27	1687.65	14.16	18.10	18852.50
273	Pailani	25.76	80.42	105	856.35	1835.60	15.03	19.33	19158.33
274	Palana	27.84	73.26	268	688.27	1568.55	15.54	19.73	18815.00
275	Pali	25.77	73.32	214	912.70	2039.25	16.97	21.03	19098.33
276	Palitana	21.53	71.82	57	1000.73	2016.65	17.97	20.84	19039.17
277	Palwal	28.14	77.33	196	713.88	1399.25	14.18	18.03	18090.00
278	Panchgani	17.92	73.79	1262	1047.11	1787.75	17.02	18.93	18494.17
279	Panipat	29.39	76.96	243	714.30	1550.75	15.59	19.55	18138.00
280	Parbatsar	26.88	74.76	428	886.21	1847.00	15.64	19.43	18695.00
281	Pariat	23.23	80.13	431	809.55	2027.10	20.82	23.53	19341.35
282	Parlakimedi	18.77	84.09	74	809.55	2027.10	20.82	23.53	19341.35
283	Patan	17.37	73.89	576	911.25	1988.70	16.87	20.61	19201.67
284	Patna	25.59	85.13	46	856.16	1754.55	16.03	19.56	19241.59
285	Patti	25.91	82.19	85	874.93	1773.35	14.75	18.81	19280.83
286	Pauta	29.18	78.47	219	925.89	1787.20	15.73	18.93	18158.33
287	Pawata	27.35	74.65	355	899.65	1833.50	15.69	19.31	19163.33
288	Pawayan	27.08	80.68	128	883.69	1600.45	13.78	17.37	18043.33

289	Phalodi	26.72	73.92	320	597.09	1237.35	14.86	19.14	18490.24
290	Pharenda	27.1	83.27	77	873.35	1772.70	14.67	18.81	18750.00
291	Phulpur	25.55	82.08	88	875.60	1771.50	14.74	18.80	19351.67
292	pilibhit City	28.62	79.81	176	926.09	1773.95	15.66	18.82	18263.33
293	Pisangan	26.39	74.37	392	891.12	1810.25	15.32	19.12	19093.33
294	Pratapgarh	25.89	81.94	88	909.24	1836.75	15.51	19.34	19255.00
295	Punahana	27.86	77.2	192	891.65	1818.25	15.60	19.19	18356.67
296	Pupri	26.46	85.69	43	873.05	1799.85	15.19	19.03	19045.83
297	Puranpur	28.51	80.14	181	921.45	1694.85	14.55	18.16	18256.67
298	Purwa	26.45	80.77	113	888.13	1834.35	15.39	19.32	18987.50
299	Raebareli	26.23	81.24	108	892.26	1828.50	15.23	19.27	19155.83
300	Raghapur	25.53	85.38	37	892.87	1856.60	15.81	19.51	19326.67
301	Raisingnagar	29.53	73.44	160	580.60	1333.75	16.68	20.52	18443.21
302	Rajaund	29.57	76.48	228	831.84	1846.20	15.27	19.42	18114.17
303	Rajgarh1	28.71	76.09	210	859.47	1996.80	16.71	20.67	18248.33
304	Ramban	26.51	85.09	51	738.31	1429.00	14.65	17.39	17972.90
305	Ramgarh	23.63	85.51	323	881.89	1853.80	15.31	19.48	18391.67
306	Ramgiri	17.05	79.27	223	1005.70	1794.40	16.19	18.99	18646.67
307	Ramnagar1	27.1	84.19	87	911.80	1747.35	14.76	18.59	18474.17
308	Ramnagar3	32.48	75.19	792	606.78	1253.70	16.44	19.34	19029.27
309	Rangiya	26.43	91.62	44	954.87	1931.00	16.74	20.13	19257.50
310	Ratangarh	28.06	74.61	305	576.28	1175.40	13.93	18.38	18073.17
311	Rath	25.6	79.56	146	847.82	1835.65	15.00	19.33	19350.00
312	Rewari	28.19	76.62	236	723.12	1495.20	15.66	19.30	18765.31
313	Rohtak	28.89	76.6	224	803.35	1733.80	16.07	19.80	18686.36
314	Roorkee	29.85	77.88	272	865.32	1674.55	14.37	17.99	17658.33
315	Rudrapur	28.98	79.41	197	887.59	1623.90	14.11	17.57	17820.83
316	Sabour	25.24	87.04	35	896.14	1901.90	16.03	19.88	19297.50
317	Sadabad	27.43	78.03	176	881.13	1748.95	14.96	18.61	18726.67
318	Safipur	26.73	80.34	124	592.13	1140.60	13.99	17.96	18690.24
319	Saharanpur	29.96	77.55	281	877.32	1622.70	14.17	17.56	17948.33
320	Sahaswan	28.07	78.75	164	852.88	1689.50	14.11	18.11	18160.83
321	Saidpur Bhitari	25.55	83.25	67	877.27	1768.80	14.72	18.77	19310.00
322	Salimpur	25.61	85.14	49	770.70	1454.55	13.85	17.63	18922.43
323	Salon	26.03	81.45	93	860.55	1787.50	14.86	18.93	19110.00
324	Sambhal	28.59	78.57	195	714.49	1377.00	14.06	17.81	17990.00
325	Sambhar	26.9	75.18	364	841.59	1751.15	14.40	18.63	18944.17

326	Samodh	24.38	84.51	203	768.37	1614.20	15.11	18.98	18820.37
327	Sampla	28.77	76.77	217	849.19	1711.45	14.28	18.30	18024.17
328	Sandila	27.07	80.51	132	840.91	1671.10	14.02	17.96	18695.83
329	Sanehighar	26.79	81.54	99	886.23	1828.80	15.34	19.27	19035.00
330	Sanganer	26.82	75.77	386	886.23	1828.80	15.34	19.27	19035.00
331	Sapotra	26.29	76.74	252	870.27	1792.55	14.88	18.97	18961.67
332	Saraon	25.03	84.26	87	907.99	1842.65	15.54	19.39	19325.83
333	Sardarshahr	28.43	74.49	253	676.09	1534.65	15.48	19.70	18498.98
334	Sardhana	29.14	77.61	227	868.41	1631.90	14.08	17.63	18061.67
335	Sarila	25.77	79.67	131	855.38	1811.75	14.78	19.13	19353.33
336	Sawai Madhopur	26.03	76.35	255	868.74	1855.55	15.25	19.50	19310.83
337	Shahabad	27.64	79.94	141	866.05	1676.45	14.23	18.00	18234.17
338	Shahdhra	28.68	77.26	209	861.40	1727.55	14.65	18.43	18160.83
339	Shahganj	27.17	77.97	176	871.08	1761.00	14.60	18.71	19037.50
340	Shahjahanpur	27.88	79.91	151	910.45	1832.05	15.71	19.30	18666.67
341	Shahpur	23.19	77.42	491	871.08	1761.00	14.60	18.71	19037.50
342	Sheo	26.18	71.24	234	867.10	1919.60	15.79	20.03	18873.33
343	Sheohar	26.51	85.29	56	896.05	1821.95	15.41	19.22	18864.17
344	Sheopur	25.67	76.69	234	873.76	1844.45	15.17	19.40	19205.00
345	Shergarh	26.35	72.23	249	872.67	2012.75	16.85	20.81	18963.33
346	Shikohabad	27.1	78.58	162	630.06	1298.55	16.79	20.08	18875.31
347	Shivpuri	25.43	77.66	465	853.50	1873.65	15.79	19.65	19405.00
348	Sibsagar	26.98	94.64	87	905.13	1775.20	15.53	18.83	17920.00
349	Sidhauli	27.28	80.83	124	888.51	1764.10	14.94	18.73	18517.50
350	Sikandarabad	28.45	77.69	206	705.90	1371.70	14.04	17.90	18002.02
351	Sikandarpur	26.03	84.03	52	877.75	1847.05	15.34	19.43	18926.67
352	Sikandra Rao	27.68	78.37	170	871.48	1705.20	14.47	18.24	18717.50
353	Sikar	27.6	75.13	435	731.47	1625.10	15.12	19.37	18984.91
354	Silchar	24.83	92.77	11	906.18	2045.40	17.53	21.08	19287.50
355	Siliguri	26.72	88.39	109	887.63	1950.40	16.46	20.29	19129.17
356	Sirathu	25.64	81.32	106	884.96	1735.75	14.54	18.50	19049.17
357	Sirmaur	30.56	77.47	1007	863.62	1786.65	14.73	18.92	19385.00
358	Sirsa	29.53	75.02	192	837.87	1802.35	14.76	19.05	17631.67
359	Sitapur	27.58	80.67	127	890.71	1786.35	15.13	18.92	18451.67
360	Siwana	28.7	76.43	209	912.62	2085.65	17.42	21.41	19155.00
361	Siwani	28.9	75.61	201	857.91	2014.35	16.89	20.82	18183.33

362	Sodam	13.55	78.91	528	1048.88	2041.05	18.72	21.04	18201.67
363	Sohana	30.25	77.02	274	884.36	1810.55	15.66	19.12	18237.50
364	Sojat	25.92	73.66	278	873.44	1930.00	15.97	20.12	19157.50
365	Soraon	25.6	81.84	96	907.99	1842.65	15.54	19.39	19325.83
366	Sri Madhopur	27.47	75.59	491	819.45	1645.30	14.81	18.73	19154.46
367	Sujangarh	27.7	74.46	310	792.95	1681.20	15.33	19.32	19075.45
368	Sultanpur	26.26	82.07	98	877.80	1779.90	14.81	18.87	18895.00
369	Suratgarh	29.32	73.9	167	580.76	1339.40	16.77	20.59	18501.23
370	Tanakpur	29.07	80.1	253	900.59	1598.65	14.17	17.36	17640.00
371	Tanda	26.54	82.66	75	885.97	1827.10	15.43	19.26	18870.83
372	Tarabganj	26.94	81.99	90	684.23	1395.75	15.37	19.21	18583.70
373	Tauru	28.21	76.95	266	882.71	1821.35	15.65	19.21	18191.67
374	Tezpur	26.65	92.79	70	942.14	1824.25	15.91	19.24	19100.83
375	Thakurdwara	29.19	78.86	224	895.58	1801.50	15.76	19.05	17745.83
376	Thalli	12.58	77.65	898	1030.75	2147.20	19.76	21.93	19041.67
377	Tijara	27.93	76.85	295	857.63	1794.10	14.83	18.98	18422.50
378	Todabhim	26.91	76.81	251	873.71	1874.50	15.60	19.65	18921.67
379	Tohana	29.71	75.9	226	898.25	1955.45	16.47	20.33	19228.33
380	Tonk	26.16	75.78	281	898.25	1955.45	16.47	20.33	19228.33
381	Tufanganj	26.33	89.66	32	926.56	1931.50	16.47	20.13	19113.33
382	Tura	25.51	90.2	294	969.53	1840.65	16.21	19.37	18242.50
383	Unnao	26.53	80.48	120	883.68	1865.65	15.75	19.58	18786.67
384	Vayittiri	11.55	76.04	750	1052.98	1959.75	18.24	20.36	18730.83
385	Vembavur	10.29	78.34	840	1065.19	2007.25	18.54	20.76	19133.33
386	Zamana	25.26	83.34	88	875.83	1771.65	14.71	18.80	19347.50

Class #2 (Locations: 58)

SrNo	Station	Lat	Long	Alt	P days (7,21,30)	GDD (4 deg C base temp)	Mean Night temp	Mean T	Mean radn (Kj/day/m2)
1	Amalapuram	16.57	82.00	4	631.31	1455.95	15.17	19.21	16370.83
2	Ambala	30.37	76.77	274	631.31	1455.95	15.17	19.21	16370.83
3	Ambalaa	30.23	76.46	272	631.07	1456.30	15.17	19.21	16368.75
4	Anupgarh	29.18	73.19	140	583.57	1273.95	14.37	18.52	16629.55
5	Askote	29.77	80.34	1291	909.13	1763.90	16.31	18.73	16665.00
6	Bazpur	29.15	79.14	221	926.80	1757.85	15.38	18.68	17356.67
7	Bhogpur	30.25	78.23	681	845.99	1576.65	14.94	17.87	17029.82
8	Chenani	33.04	75.29	1101	713.84	1371.35	14.47	17.34	17519.42
9	Chikodi	16.43	74.59	640	859.21	1924.15	20.00	22.72	16961.17
10	Dadupur	29.77	76.97	249	856.47	1703.10	14.49	18.23	17469.17
11	Dehradun	30.32	78.03	648	802.41	1491.85	14.78	17.85	17140.74
12	Dibrugarh	27.47	94.91	95	917.59	1730.05	15.63	18.70	16670.34
13	Gadarpur	29.04	79.25	201	922.92	1748.35	15.24	18.60	17412.50
14	Gulah	27.74	79.45	137	594.16	1126.40	13.92	17.79	17408.54
15	Haldwani	29.22	79.51	413	930.82	1611.20	14.55	17.46	17375.00
16	Imphal	24.82	93.94	774	795.28	1536.45	15.50	18.81	17307.69
17	Jejuri	18.28	74.16	729	978.28	2218.05	20.00	22.52	16940.00
18	Junnar	19.20	73.87	684	1022.65	2238.20	20.29	22.69	16922.50
19	Kalsia	30.63	75.68	238	870.54	1722.10	15.02	18.38	17406.67
20	Kangra	32.10	76.27	771	899.14	1514.05	14.30	16.65	16620.83
21	Karanpur	30.33	78.05	686	791.08	1653.15	13.50	17.81	17682.50
22	Karnaprayag	30.26	79.22	742	887.91	1632.65	14.97	17.64	17003.33
23	Katbari	22.22	86.01	306	923.43	1943.60	16.58	16.74	16703.45
24	Kathghora	22.50	82.55	314	942.92	2116.20	18.36	18.18	16719.58
25	Kathgodam	29.27	79.54	524	954.40	1885.45	17.28	19.75	17742.50
26	Kathua	32.39	75.52	340	759.86	1569.60	15.23	18.57	16841.67
27	Kharar	30.75	76.64	303	633.18	1434.40	15.04	18.98	16181.25
28	Kotdwara	29.75	78.53	397	970.51	1854.15	17.13	19.48	15707.50
29	Kumily	9.61	77.17	884	1042.26	2151.75	19.78	21.96	17497.50
30	Landsdown	29.84	78.69	1713	865.62	1616.75	16.19	18.60	16188.29
31	Ludhiana	30.90	75.86	247	549.88	1025.10	12.53	16.70	17012.35
32	Madhopur	32.37	75.60	353	892.65	1747.25	14.97	18.59	16212.50
33	Mahrauli	31.25	76.46	308	662.16	1446.45	15.04	18.76	16503.06
34	Mandya	12.52	76.90	665	466.99	1089.30	21.35	23.52	17339.29

35	Mat	29.64	79.65	1729	638.44	1484.60	15.39	19.46	17347.92
36	Mount Abu	24.59	72.71	1189	914.28	1768.80	15.91	18.77	17775.00
37	Muhammadabad	26.03	83.38	67	885.45	1806.35	15.07	15.91	16047.92
38	Murwara	23.83	80.4	366	912.93	1926.75	16.36	16.18	15997.99
39	Muzaffarnagar	29.47	77.7	239	889.06	1749.20	15.19	18.01	17202.42
40	Nakodar	31.12	75.48	226	668.99	1545.20	15.14	19.19	16290.20
41	Nakur	29.91	77.3	263	862.89	1671.80	14.43	17.97	17344.17
42	North Lakhimpur	27.22	94.1	77	955.08	1787.95	15.75	18.93	17136.67
43	Palampur	32.11	76.53	1257	874.41	1608.00	15.37	17.43	16281.67
44	Panki	26.46	80.24	120	921.18	1732.10	16.09	18.47	16653.33
45	Partapganj	26.29	86.96	60	881.51	1873.90	15.81	15.82	15471.81
46	Pathankot	32.26	75.64	320	802.65	1547.25	14.74	18.10	17471.82
47	Patiala	30.33	76.38	252	633.45	1474.90	15.38	19.41	16351.04
48	Peraiyur	9.73	77.7	168	1040.74	1791.80	17.01	18.97	16774.17
49	Raipur1	26.11	80.8	108	892.48	1564.45	14.21	17.07	16857.50
50	Rajpur	30.38	78.08	883	878.36	1625.85	14.80	17.58	16747.50
51	Ramnagar	29.39	79.12	350	924.36	1674.80	15.25	17.99	17252.50
52	Reasi	33.08	74.83	527	794.82	1493.20	13.25	16.48	16333.33
53	Samba	31.95	76.5	761	822.85	1498.30	13.37	16.52	16351.67
54	Sidlaghatta	13.39	77.86	897	978.20	2300.55	20.91	23.20	17606.67
55	Sri Ranbhirsinghpura	32.65	74.83	260	822.13	1537.40	13.53	16.85	16511.67
56	Srinagar_UK	34.08	74.79	1579	902.81	1541.65	14.29	16.88	17133.33
57	Tibri	32	75.45	263	738.43	1527.75	14.80	18.32	17047.66
58	Uthamapalayam	9.8	77.32	375	469.75	1191.55	21.89	24.26	17744.07

Class #3 (Locations: 540)

Sr No	Station	Lat	Long	Alt	P days (7,21,30)	GDD (4 deg C base temp)	Mean Nifgt temp	Mean T	Mean radn (Kj/day/m2)
1	Amalner	18.94	75.33	698	400.04	1026.20	18.99	23.08	21255.56
2	Amarpur	25.03	86.90	60	941.35	2115.35	18.13	21.66	20117.50
3	Amarwara	22.29	79.16	809	921.47	1704.00	14.63	18.23	20104.17
4	Ambagarh Chowki	20.77	80.74	325	579.41	1197.65	16.46	20.24	20651.35
5	Ambari	30.49	77.81	502	579.41	1197.65	16.46	20.24	20651.35
6	Ambikanagar	15.12	74.65	497	929.12	2077.35	17.81	21.34	20289.17
7	Amraoti	21.64	78.18	694	913.26	2144.90	18.10	21.91	20840.00
8	Anand	22.56	72.92	34	832.65	2234.50	18.00	22.65	20584.17
9	Anandpur	21.21	86.12	42	685.36	1697.70	19.05	22.70	20765.93
10	Angul	20.84	85.15	109	799.54	2021.70	19.09	22.76	21011.11
11	Anjangaon	18.08	75.60	481	937.45	1990.45	17.16	20.62	20391.67
12	Anjar	23.11	70.02	64	463.96	1130.85	17.06	21.46	19709.23
13	Antagarh	20.09	81.15	363	731.45	1714.25	17.33	21.36	21137.37
14	Arambagh	22.87	87.79	12	876.66	2197.15	18.65	22.34	20376.67
15	Arang	21.19	81.96	277	905.86	2234.65	19.04	22.66	20512.50
16	Armoor	18.79	78.29	371	878.18	2295.65	19.92	23.49	20668.64
17	Arogyavaram	18.94	84.58	20	994.97	2225.15	19.86	22.58	20075.83
18	Arvi	20.63	79.14	260	771.27	1998.60	19.98	23.44	20400.00
19	Asansol	23.67	86.95	117	723.20	1715.75	18.36	22.10	19643.16
20	Asifabad	19.36	79.29	201	440.68	1000.95	19.35	22.61	21255.56
21	Aslali	22.91	72.59	37	863.67	2163.05	17.67	22.06	20352.50
22	Asola	28.46	77.19	259	898.79	2224.90	18.77	22.57	21002.50
23	Astha	23.19	77.44	478	894.19	2002.65	16.68	20.72	20011.67
24	Atmakur	15.88	78.59	286	677.72	1679.40	20.81	23.57	20838.37
25	Atner	21.62	77.91	661	922.53	2014.05	17.18	20.82	19976.67
26	Atru	24.88	76.67	284	841.72	1978.35	16.00	20.52	19642.50
27	Aurangabad	19.88	75.34	594	715.11	1820.80	17.84	22.25	20994.00
28	Azimganj	24.23	88.25	14	903.92	2074.15	17.49	21.32	19985.00
29	Badnagar	23.03	75.37	500	888.19	2045.95	16.97	21.08	20009.17
30	Badnera	20.85	77.73	324	904.18	2161.95	18.19	22.05	20738.33

31	Bagodar	24.05	85.83	383	953.56	1843.55	16.03	19.40	19670.83
32	Bahoribund	23.66	80.06	421	925.77	1893.15	16.21	19.81	19894.17
33	Baihar	22.10	80.54	546	875.77	1788.45	15.01	18.94	20084.17
34	Bajag	22.67	81.35	780	901.21	1641.00	14.46	17.82	19959.66
35	Bakani	22.47	86.59	34	898.79	1915.25	15.87	19.99	19781.67
36	Balapur	19.53	77.41	417	570.08	1595.55	20.17	23.99	20216.25
37	Balasinor	22.95	73.33	86	439.84	1301.35	17.93	22.86	20827.54
38	Balasore	21.48	86.92	17	585.63	1382.90	18.32	22.01	20467.53
39	Balliguda	20.19	83.00	540	950.34	1891.85	16.67	19.80	20168.33
40	Balodabazar	21.65	82.10	252	913.43	2231.35	18.91	22.63	20691.67
41	Balumath	23.82	84.78	546	900.79	1715.80	14.58	18.33	19895.83
42	Bankura	23.23	87.07	72	904.17	2117.50	18.10	21.68	19993.33
43	Bano	22.64	84.92	478	955.97	1974.95	17.23	20.49	19930.00
44	Banswara	23.54	74.43	211	478.67	1255.50	19.73	23.38	20186.15
45	Baraset	22.43	88.29	6	864.24	2178.60	18.71	22.50	20633.05
46	Barerakalan	23.06	80.02	428	913.05	1864.25	15.86	19.57	19973.33
47	Barhi	24.30	85.41	376	760.43	1692.80	17.47	21.14	19566.67
48	Bari	26.39	77.36	191	806.09	1684.45	15.52	19.35	19406.36
49	Baripada	21.93	86.75	40	909.36	2177.45	18.62	22.18	20480.83
50	Barjora	23.42	87.28	77	915.79	2107.40	17.94	21.60	20016.67
51	Barrachpore	22.76	88.38	6	705.23	1689.85	18.19	22.02	20081.91
52	Basirhat	22.65	88.86	8	856.26	2194.30	18.87	22.63	20464.41
53	Basoda	23.85	77.92	419	924.44	2049.05	17.16	21.11	20130.83
54	Begumpet	17.44	78.46	518	929.94	2265.05	19.72	22.91	20822.50
55	Belathal	24.70	73.97	490	857.32	1808.85	14.68	19.11	19620.83
56	Belonia	23.25	91.46	27	590.25	1304.95	18.54	21.93	19394.52
57	Bemetara	21.71	81.53	284	865.66	2004.50	18.18	21.77	20353.10
58	Berhampore	24.09	88.26	14	884.88	2072.10	17.51	21.30	19859.17
59	Berhampur	19.31	84.79	26	684.51	1671.75	20.27	23.26	20628.74
60	Betul	21.91	77.90	650	918.45	2019.60	17.18	20.86	19877.50
61	Bhadgaon	20.66	75.22	256	432.23	1126.25	17.98	22.53	21059.02
62	Bhadrachalam	17.66	80.89	71	763.24	2254.25	21.39	24.72	21097.25
63	Bhadrak	21.05	86.49	12	583.90	1393.50	18.41	22.15	20509.09
64	Bhagwanpur	26.12	85.36	50	864.12	2231.10	19.36	22.94	20520.34

65	Bhakarapet	14.41	78.95	136	1026.31	2081.80	18.84	21.38	20372.50
66	Bhandara	21.17	79.65	248	872.46	2065.10	17.88	21.84	20888.79
67	Bhanupratappur	20.29	81.07	353	897.79	2063.40	17.33	21.23	20938.33
68	Bhatagaon	21.21	81.62	279	717.38	1757.60	18.13	22.16	20610.31
69	Bhawanipatna	19.90	83.16	249	880.46	2211.15	19.41	22.93	20962.39
70	Bhillai	21.19	81.35	287	894.44	2054.80	17.87	21.60	20811.11
71	Bhilsa	23.52	77.80	429	928.32	2035.70	17.09	21.00	20183.33
72	Bhind	26.55	78.78	142	802.23	1697.85	15.62	19.47	19430.91
73	Bhinmal	25.00	72.26	156	881.62	2244.40	18.87	22.74	19533.33
74	Bhopal	23.25	77.41	484	703.15	1964.20	19.63	23.68	19603.00
75	Bhubneshwar	20.29	85.82	33	546.58	1425.05	20.01	23.58	20478.08
76	Bhuj	23.24	69.66	100	476.41	1187.05	17.45	21.78	19911.94
77	Bhungra	23.41	74.31	244	847.62	2191.30	18.13	22.45	20032.77
78	Bhusaval	21.04	75.80	209	466.93	1148.30	18.20	22.29	20811.11
79	Bichhia	22.45	80.71	628	871.55	1671.95	13.88	17.97	19915.83
80	Bidar	17.91	77.05	662	786.52	1959.65	19.38	22.88	20689.42
81	Bijanagar	25.92	74.65	398	823.34	1739.20	14.03	18.53	19517.50
82	Bikaunthpur	23.27	82.55	554	963.78	1937.75	17.06	20.18	19774.17
83	Bishnupur	23.06	87.31	71	931.81	1752.80	15.51	18.64	19558.33
84	Bobbili	18.57	83.35	127	721.53	1833.05	20.43	23.54	20477.66
85	Bodalkasa	21.75	79.79	217	902.33	2196.80	18.54	22.34	21142.50
86	Boinchee	23.12	88.19	5	740.86	1739.90	17.87	21.62	20044.44
87	Bolpur	23.66	87.68	46	749.11	1723.65	17.71	21.45	19939.39
88	Bongaon	23.04	88.82	9	851.24	2179.00	18.29	22.34	20547.06
89	Bonth	21.12	86.30	25	578.43	1389.10	18.56	22.33	20588.16
90	Bori	20.91	79.01	240	949.94	1983.85	16.91	20.57	20477.50
91	Borina	25.06	76.39	270	924.25	1943.95	16.60	20.23	19853.33
92	Borsad	22.41	72.89	27	477.72	1226.35	19.01	22.93	20116.92
93	Budhana	29.28	77.47	231	890.12	1777.70	15.43	18.85	17838.33
94	Burdwan	23.23	87.86	27	929.37	2104.65	18.14	21.57	19999.17
95	Burhanpur	21.31	76.22	253	759.77	1928.85	18.55	22.59	20634.62
96	Chaibasa	22.55	85.80	233	856.37	1907.20	17.44	21.06	20335.71
97	Chakaibanda	24.32	86.24	290	902.56	1837.35	15.74	19.34	19495.83
98	Chakradhanpur	22.68	85.63	224	871.16	1527.90	14.08	17.09	19637.61

99	Chalisgaon	20.46	75.00	349	822.93	2196.05	18.72	22.97	21137.93
100	Champa	22.03	82.65	247	580.79	1307.75	17.96	21.73	20198.65
101	Champua	22.06	85.67	386	921.33	1855.25	15.75	19.49	20365.83
102	Chandbali	20.77	86.74	6	889.43	1921.75	16.20	20.05	19578.33
103	Chanditala	22.69	88.27	7	902.93	2161.80	18.30	22.05	20675.83
104	Chandpur	29.14	78.27	226	906.95	2236.65	19.10	22.67	20647.50
105	Chandrakona	22.73	87.52	35	784.02	1982.85	18.42	22.40	20568.52
106	Channagiri	14.02	75.93	671	946.41	2225.30	19.33	22.58	20992.50
107	Charchal	25.20	87.55	33	889.43	1921.75	16.20	20.05	19578.33
108	Charkhari	25.40	79.75	185	827.04	1767.80	14.27	18.77	19495.00
109	Chaurai	22.05	79.25	639	882.09	1904.45	15.76	19.90	20369.17
110	Chicholi	21.49	79.71	303	941.62	1923.70	16.47	20.06	20249.17
111	Chikhalda	21.40	77.33	1079	914.14	1758.30	15.27	18.69	20067.50
112	Chikhli	20.76	73.06	24	656.83	1838.70	20.16	24.03	20891.30
113	Chimur	20.50	79.37	240	753.05	1756.75	17.58	21.61	20597.00
114	Chindwara	22.06	78.94	676	933.54	1920.05	16.40	20.03	20225.83
115	Chintakunta	17.29	77.79	525	676.03	1797.90	20.44	23.80	21242.86
116	Chitradurg	14.23	76.40	713	812.84	1997.30	20.66	23.43	20429.13
117	Chodavaram	17.83	82.93	42	606.80	1447.20	20.73	23.35	19644.00
118	Chopda	21.25	75.30	188	401.33	1003.00	18.48	22.65	21224.07
119	Chorkhamara	21.27	79.96	306	909.57	2223.25	18.91	22.56	20740.83
120	Chotan	26.85	80.95	112	682.06	1475.75	16.71	20.44	19482.22
121	Chuda	22.48	71.69	70	844.25	2068.70	16.76	21.27	20577.50
122	Cuttack	20.46	85.88	26	540.50	1423.65	19.88	23.56	20658.90
123	Damoh	23.84	79.44	377	736.91	1565.05	16.50	20.34	19790.63
124	Danpur	25.27	83.26	69	878.71	2094.40	17.18	21.49	20322.50
125	Dansa	22.53	88.87	12	769.35	1924.30	18.62	22.54	20802.88
126	Dantan	21.91	87.27	14	931.61	2195.35	18.96	22.33	20239.17
127	Dantewara	18.85	81.38	385	896.01	2222.80	18.88	22.56	21032.50
128	Darwha	20.31	77.77	330	749.11	1850.25	17.95	22.18	20915.69
129	Daryapur	20.92	77.33	270	769.35	1924.30	18.62	22.54	20802.88
130	Datia	25.67	78.46	252	640.04	1274.85	14.89	18.53	19477.27
131	Deesa	24.26	72.19	141	848.78	2138.65	17.57	21.86	19731.67
132	Deobhog	19.90	82.66	247	520.78	1388.45	18.72	23.07	21228.77

133	Deogarh	21.54	84.73	200	905.48	2007.25	17.62	21.19	20386.32
134	Deolapur	26.66	82.74	80	927.57	2149.60	18.38	21.95	20616.67
135	Deolgaonraja	20.63	75.99	393	745.67	1932.75	18.40	22.62	20530.77
136	Deori	23.39	79.02	429	884.72	2091.75	18.20	21.91	20316.24
137	Deuli	22.08	87.33	17	742.85	1805.70	18.60	22.28	19909.09
138	Dewas Senior	22.96	76.05	532	886.72	1977.70	16.37	20.51	20194.17
139	Dhamangaon	20.79	78.14	293	429.25	1267.95	20.56	24.52	20430.65
140	Dhambola	23.59	73.77	220	486.99	1138.55	17.49	21.58	19769.23
141	Dhanbad	23.80	86.43	249	920.65	2031.20	17.44	20.96	19828.33
142	Dhanora	22.38	73.10	23	719.53	1668.95	17.71	21.61	20745.26
143	Dhanwar	24.41	85.98	341	930.43	1880.90	16.17	19.71	19461.67
144	Dharamanagar	24.38	92.15	17	577.77	1269.60	17.43	21.21	19256.76
145	Dharamjaigarh	22.46	83.21	299	690.83	1748.20	17.92	22.06	20849.48
146	Dharamsala	32.22	76.32	1321	559.91	1357.65	18.97	22.65	20678.08
147	Dharni	21.55	76.89	310	861.88	2232.20	18.28	22.64	20969.17
148	Dhond	18.65	75.31	568	870.53	2304.20	19.38	23.24	20957.50
149	Dhoraji	21.74	70.45	55	838.28	2354.05	19.73	23.65	20590.83
150	Dhrangadhra	22.98	71.47	60	846.75	2215.90	18.13	22.50	20410.83
151	Dhuti	22.12	80.06	304	594.20	1339.40	17.78	21.45	19977.92
152	Diamond Harbour	22.20	88.20	5	594.20	1339.40	17.78	21.45	19977.92
153	Digras	18.41	76.95	624	741.32	1868.65	18.07	22.36	20971.57
154	Dindori	20.20	73.83	625	994.53	1962.60	17.29	20.39	19935.00
155	Dindoria	22.94	81.08	667	923.80	1951.20	16.53	20.29	20124.17
156	Dinhata	26.13	89.47	33	923.80	1951.20	16.53	20.29	20124.17
157	Donegaon	20.11	76.43	571	938.93	2055.15	17.53	21.16	20550.83
158	Dongargaon	20.97	80.85	305	611.16	1352.60	16.64	20.75	20637.04
159	Dudhi	24.22	83.24	239	923.53	1804.05	15.30	19.07	19810.83
160	Dug	23.93	75.83	495	888.77	2054.45	17.34	21.15	19760.83
161	Dumdum	22.65	88.43	8	865.82	2175.75	18.72	22.47	20589.83
162	Dumri	24.00	86.00	274	947.64	2037.20	17.65	21.01	19633.33
163	Dungarpur	23.84	73.71	287	874.54	1979.35	16.38	20.53	19935.83
164	Durg	21.19	81.28	297	890.65	2061.80	17.88	21.66	20816.24
165	Dwarka	22.24	68.97	6	890.65	2061.80	17.88	21.66	20816.24
166	Edalabad	21.05	76.06	215	489.68	1206.30	18.71	22.62	20549.23

167	Gadarwara	22.92	78.78	333	937.52	2037.95	17.43	21.02	20135.00
168	Gandai	21.67	81.10	327	883.55	2044.25	17.55	21.51	20838.46
169	Gangajalghati	23.42	87.11	109	902.66	2099.85	17.93	21.53	19966.67
170	Ganganagar	29.90	73.88	169	419.60	966.80	17.51	21.34	19794.64
171	Gannavaram	16.54	80.80	13	432.99	1193.90	21.63	24.65	21324.14
172	Gargoti	16.32	74.14	556	916.65	2330.00	20.20	23.45	20242.50
173	Garhakota	23.78	79.14	388	956.48	1963.70	16.92	20.40	20116.67
174	Gariabund	20.63	82.06	336	909.12	2163.50	18.42	22.06	20304.17
175	Garotha	25.92	82.63	76	822.14	1668.70	13.58	17.94	19454.17
176	Garothaa	24.20	75.39	411	910.75	1937.75	16.23	20.18	19710.00
177	Gattasili	20.45	81.80	401	929.55	1969.05	16.77	20.44	20610.83
178	Gazole	25.21	88.19	42	919.49	1910.15	16.18	19.95	19531.67
179	Ghansore	22.66	79.94	553	912.29	1954.30	16.72	20.32	19940.00
180	Ghatal	22.66	87.75	10	874.42	2208.25	18.74	22.44	20403.33
181	Gohad	26.45	78.43	157	809.53	1709.15	15.59	19.43	19477.48
182	Gondal	21.96	70.79	132	919.30	2130.95	18.04	21.79	20696.67
183	Gondia	21.46	80.22	293	591.14	1359.00	18.56	21.93	20319.74
184	Gopalpur	19.26	84.86	19	750.30	1810.30	20.06	23.10	20569.47
185	Gosaba	22.17	88.81	8	889.37	2231.90	19.08	22.63	20482.50
186	Guna	24.63	77.30	485	898.30	1843.65	15.35	19.40	19582.50
187	Guntur	16.31	80.44	19	431.51	1162.25	21.46	24.46	21187.72
188	Gwalior	26.22	78.18	226	707.21	1529.35	14.95	19.03	19326.47
189	Harda	22.34	77.10	283	892.75	2160.30	17.90	22.04	20565.00
190	Harduamarar	23.46	79.33	366	938.07	1924.80	16.50	20.07	20055.00
191	Harrai	22.62	79.22	576	912.70	1829.15	15.45	19.28	20433.33
192	Harsud	22.10	76.74	260	475.87	1194.75	18.40	22.44	20718.46
193	Hatta	24.13	79.60	322	906.09	1979.40	16.67	20.53	19675.00
194	Hazaribagh	24.00	85.37	601	922.70	1754.95	15.24	18.66	19547.50
195	Himatnagar	23.60	72.96	140	890.56	2045.60	16.98	21.08	20010.00
196	Himayat Sagar	17.31	78.36	544	783.22	1895.70	19.13	22.62	20664.71
197	Hinganghat	20.55	78.84	222	471.15	1173.80	18.18	22.40	20870.31
198	Hingoli	19.72	77.15	459	837.32	1996.15	18.57	22.18	20237.27
199	Hiwarkhed	21.13	76.86	313	741.93	1984.75	20.30	23.69	20630.69
200	Hoshangabad	22.74	77.74	305	923.77	2162.40	18.47	22.05	20198.33

201	Howarh	22.60	88.26	4	903.06	2155.70	18.32	22.00	20160.00
202	Huzurabad	18.20	79.40	260	750.66	1971.05	20.06	23.55	20973.27
203	Igatpuri	19.70	73.56	584	1006.98	1970.85	17.67	20.46	20870.00
204	Iklera	25.02	76.54	270	867.97	1968.30	16.15	20.44	19840.00
205	Indore	22.72	75.86	538	842.85	1977.45	15.98	20.51	20365.00
206	Indpur	23.16	86.94	128	724.20	1706.90	18.26	22.01	19778.95
207	Indus	23.09	87.38	39	751.88	1747.15	18.03	21.69	20009.09
208	Itahar	25.45	88.17	25	914.08	1941.85	16.61	20.22	19565.83
209	Jabalpur	23.18	79.99	411	896.41	1840.70	15.63	19.37	19812.50
210	Jabera	23.56	79.68	347	867.29	1962.70	16.20	20.39	20105.83
211	Jactial	18.79	78.91	285	697.15	1959.80	19.91	23.84	20895.96
212	Jagastsingpur	20.25	86.17	9	702.91	1863.65	20.27	23.66	20529.47
213	Jagpura	25.06	75.87	334	864.63	2140.70	17.57	21.87	20262.50
214	Jaipur	26.91	75.79	430	917.55	1983.70	16.99	20.56	19896.67
215	Jalgaon	21.01	75.56	217	862.16	2326.00	19.35	23.42	20949.17
216	Jalore	25.34	72.63	176	897.60	2159.10	18.08	22.03	19461.67
217	Jambugoda	22.36	73.73	93	844.54	2133.60	17.24	21.81	20396.67
218	Jaminia	22.12	80.18	370	883.90	1884.05	15.54	19.73	20711.67
219	Jamnagar	22.47	70.06	17	864.89	2114.80	17.38	21.66	20445.83
220	Jamner	20.81	75.78	250	423.25	1217.20	19.74	24.02	20434.43
221	Jamshedpur	22.80	86.20	148	925.79	2077.80	17.74	21.35	20349.17
222	Jamunia	25.37	87.05	34	883.90	1884.05	15.54	19.73	20711.67
223	Janakpur	23.43	81.49	405	905.01	1887.25	15.78	19.76	20137.50
224	Janjgir	22.01	82.57	259	938.14	2197.00	18.93	22.34	20117.50
225	Jaora	23.64	75.13	471	906.82	2121.60	17.79	21.71	20025.83
226	Jashpurnagar	22.89	84.14	768	871.78	1644.80	14.03	17.74	20040.83
227	Jaso	24.50	80.52	330	903.94	1903.20	16.24	19.89	19546.67
228	Jaswantpura	24.80	72.46	278	849.04	2176.15	18.22	22.17	19776.67
229	Jayankoundam	11.21	79.36	52	491.89	1308.80	21.90	24.51	20546.88
230	Jeypore	18.86	82.55	584	967.72	1987.60	17.31	20.60	20666.67
231	Jhaiawar	24.60	76.16	315	839.16	1946.70	15.72	20.26	19695.83
232	Jhansi	25.45	78.57	250	680.96	1398.00	15.26	19.24	19184.78
233	Jharsuguda	21.86	84.01	217	736.18	1676.60	17.02	20.98	20651.52
234	Joypur	23.06	87.45	53	751.49	1730.75	17.81	21.52	20045.45

235	Junagarh	21.52	70.46	82	899.10	2219.60	18.78	22.53	20826.67
236	Kaira	22.45	72.42	36	834.76	2250.15	18.21	22.78	20525.00
237	Kakinada	16.99	82.25	7	358.23	939.15	21.75	24.50	20226.09
238	Kalana	28.50	75.30	256	866.19	2212.15	18.44	22.47	20327.50
239	Kalol	22.61	73.46	74	866.26	2168.15	17.76	22.10	20116.67
240	Kalvan	18.29	73.27	40	970.30	2033.15	17.85	20.98	19535.00
241	Kalyanganj	27.14	76.25	388	898.74	2103.85	17.76	21.57	20213.33
242	Kammareddi	18.32	78.33	509	761.00	1843.95	18.84	22.48	20448.00
243	Kanas	20.00	85.65	12	703.05	1910.40	20.66	23.94	20343.75
244	Kandi	23.95	88.05	19	899.05	2110.95	17.81	21.62	20170.83
245	Kanekal	14.81	77.07	457	634.96	1778.55	21.37	24.49	21341.38
246	Kanker	20.27	81.49	384	896.19	1929.85	16.76	20.53	20638.46
247	Kankey	23.30	85.18	621	935.36	1782.80	15.55	18.89	19594.17
248	Kanki	26.01	87.86	32	867.81	1997.65	18.13	21.71	20422.12
249	Kannod	22.66	76.74	326	450.67	1284.05	17.60	22.67	20994.20
250	Kapadvanj	23.02	73.07	77	857.15	2216.15	18.10	22.50	20303.33
251	Karimnagar	18.44	79.13	266	640.88	1687.80	20.21	23.67	20802.33
252	Karmala	18.40	75.19	555	432.29	1122.45	18.01	22.47	21239.34
253	Katangi	21.77	79.80	338	912.90	2162.40	18.20	22.05	20739.17
254	Katol	21.27	78.59	404	920.53	2143.75	18.05	21.90	20880.00
255	Kattan Jhiri	21.50	79.59	334	908.51	2173.85	18.42	22.15	20762.50
256	Katwa	23.64	88.13	8	899.42	2096.95	17.58	21.51	20548.33
257	Kawardha	22.01	81.22	360	931.99	2097.45	18.01	21.51	20245.00
258	Kendri	21.10	81.74	315	918.46	2183.90	18.61	22.23	20544.17
259	Keolari	22.37	79.91	440	952.33	2049.75	17.54	21.11	20320.83
260	Keonjhargarh	21.62	85.59	492	931.53	1829.50	15.86	19.28	20095.00
261	keshiary	22.12	87.23	36	868.35	2225.35	19.30	22.89	20573.73
262	Keskal	20.08	81.59	641	908.86	1694.25	14.68	18.15	20407.50
263	khachord	23.42	75.28	492	919.38	2058.55	17.39	21.19	19682.50
264	Khaira	21.49	86.95	12	695.89	1815.15	18.59	22.75	20611.34
265	Khairagarh	21.42	80.98	309	904.76	2047.70	17.93	21.54	20408.55
266	Khamagaon	20.71	76.57	299	658.58	1873.70	20.54	24.19	20651.61
267	Khamaria	21.80	81.33	294	878.03	2081.75	17.84	21.83	20823.93
268	Khanakul	22.73	87.87	10	874.75	2201.10	18.69	22.38	20375.00

269	Khandwa	21.83	76.35	304	725.04	1814.95	17.63	22.01	20427.72
270	Khanpur	23.03	72.58	55	890.58	1955.45	16.13	20.33	19703.33
271	Khapari	20.56	79.50	234	924.84	2177.85	18.56	22.18	20660.83
272	Kharagpore	22.35	87.23	45	579.94	1342.30	17.61	21.48	20194.81
273	Khargram	24.03	87.98	22	893.39	2108.30	17.73	21.60	19870.00
274	Kharra	21.30	81.19	286	939.54	2025.80	17.34	20.92	20719.17
275	Kharswaan	22.78	85.83	198	933.54	1987.25	16.97	20.59	20311.67
276	Khatra	22.97	86.85	126	935.01	2045.90	17.58	21.08	20296.67
277	Kherwara	23.84	73.48	325	938.55	1962.35	16.80	20.39	19561.67
278	Khilchipur	24.04	76.58	384	925.95	2112.70	17.82	21.64	20841.67
279	Khitoli	26.39	78.52	145	900.25	1891.75	16.00	19.80	19784.17
280	Kholapur	16.70	74.24	561	887.21	2192.65	18.30	22.31	20875.83
281	Khowai	24.07	91.61	14	555.87	1232.20	16.27	20.48	19417.33
282	Khurai	24.04	78.33	441	952.65	2013.75	17.28	20.81	19975.83
283	Khurja	28.25	77.85	198	952.65	2013.75	17.28	20.81	19975.83
284	Khyrburd	21.29	80.04	294	724.00	1769.05	18.31	22.28	20384.54
285	Kolaghat	22.43	87.86	8	746.11	1789.90	18.47	22.12	20271.72
286	Kondagaon	19.60	81.66	584	945.35	1957.80	16.69	20.35	21039.17
287	Kondapar	21.00	81.72	302	805.16	1915.35	18.25	21.94	20575.70
288	Konta	17.81	81.39	43	723.20	1723.00	20.04	22.98	21264.84
289	Kopargaon	19.89	74.48	504	710.84	1837.95	17.99	22.42	20886.00
290	Korapat	18.81	82.71	913	950.15	1787.25	15.80	18.93	20283.33
291	Kotalpur	23.61	87.59	33	748.56	1753.15	18.06	21.75	20033.33
292	Krishnagar	23.40	88.50	10	921.59	2115.10	17.93	21.66	19980.00
293	Kukrahati	22.18	88.12	7	593.94	1339.70	17.79	21.45	19976.62
294	Kulpahar	25.31	79.64	188	823.73	1754.75	14.14	18.66	19509.17
295	Kundahit	23.97	87.16	151	940.18	2000.50	17.18	20.70	19919.17
296	Kurdeg	22.55	84.12	417	866.18	1902.90	17.47	21.03	20083.04
297	Lakhnadon	22.60	79.60	615	907.62	1832.40	15.63	19.30	19823.33
298	Lakholi	21.20	81.90	291	911.96	2213.85	18.87	22.48	20530.83
299	Lakhpat	23.81	68.77	23	667.58	1826.05	20.25	23.89	19686.96
300	Lalbagh	12.95	77.58	893	886.11	2063.85	17.44	21.23	19840.00
301	Lalitpur	24.69	78.41	346	889.09	1813.35	15.13	19.14	19879.17
302	Lanji	21.51	80.55	307	733.16	1736.05	18.02	21.94	20350.52

303	Latehar	23.75	84.51	379	883.07	1656.15	14.03	17.83	19878.33
304	Lohardaga	23.44	84.68	651	929.70	1746.20	15.47	18.59	19620.83
305	Madanapalli	13.56	78.50	688	990.59	2238.30	19.96	22.69	20080.00
306	Madurantakam	12.51	79.88	27	355.17	955.20	22.22	24.85	20591.30
307	Mahasamund	21.06	82.06	297	867.44	1992.75	18.07	21.67	20465.49
308	Mahbubnagar	16.73	78.00	475	881.80	2255.40	20.35	23.48	20843.10
309	Maheshwar	22.12	75.58	151	878.42	2118.55	17.79	21.69	19913.33
310	Mahidpur	24.57	77.99	439	883.34	2002.30	16.49	20.72	20094.17
311	Mahoba	25.29	79.37	199	820.77	1733.00	13.96	18.48	19512.50
312	Mahroni	24.58	78.72	367	923.95	1944.70	16.59	20.24	19872.50
313	Mahudha	22.81	72.94	39	815.22	2075.80	17.83	22.24	20400.88
314	Maihar	24.26	80.75	344	691.28	1411.90	15.90	19.39	19327.17
315	Majhgahan Hansraj	24.91	80.80	330	949.01	1922.35	16.50	20.05	20175.83
316	Makrai	22.06	77.10	372	929.42	1933.30	16.27	20.14	20480.83
317	Mala	10.24	76.26	6	862.24	1974.45	16.24	20.49	20084.17
318	Malda	25.02	88.14	33	936.26	2013.30	17.26	20.81	19420.83
319	Malegaon	20.55	74.51	430	387.68	1014.00	16.14	21.55	21212.07
320	Malkapur	20.88	76.21	242	431.71	1274.15	20.82	24.62	20209.68
321	Malsiras	17.86	74.90	526	877.08	2383.25	20.44	23.89	20411.67
322	Manchappa	13.12	77.91	884	911.37	2322.75	24.31	23.39	20591.67
323	Mandhata	22.25	76.15	220	409.83	987.20	18.26	22.36	21074.07
324	Mandla	22.59	80.37	436	845.11	1728.80	14.07	18.44	20563.33
325	Mandsaur	24.07	75.06	441	876.04	1913.95	15.81	19.98	19833.33
326	Mangalkot	23.52	87.90	25	927.28	2108.35	18.15	21.60	19946.67
327	Mangrulpir	20.31	77.34	441	893.07	2159.70	18.08	22.03	20774.17
328	Manikpur	25.02	81.10	234	851.44	1784.75	14.74	18.91	19545.00
329	Mankar	23.42	87.55	63	753.62	1732.70	17.88	21.54	19935.35
330	Manoharpur	27.30	75.95	450	940.80	2036.45	17.58	21.00	20053.33
331	Manoharthana	24.53	76.17	312	745.40	1748.45	16.34	20.85	19610.58
332	Manteswar	23.42	88.10	8	899.13	2113.80	17.75	21.65	20560.00
333	Matar	22.70	72.65	27	835.51	2249.30	18.20	22.78	20532.50
334	Mau	25.94	83.54	70	902.75	1855.85	15.46	19.50	19601.67
335	Mau1	25.94	83.54	64	865.71	1813.80	14.82	19.15	19618.33
336	Mayureswar	23.98	87.76	42	749.92	1716.30	17.63	21.38	19879.80

337	Mehkar	20.14	76.57	542	778.31	1880.10	18.20	22.12	20420.19
338	Mehmadabad	22.99	72.61	57	858.95	2182.95	17.82	22.22	20372.50
339	Mehsana	23.58	72.36	77	654.41	1748.15	19.18	23.05	19993.48
340	Mhaswad	17.63	74.78	606	765.70	1914.00	19.05	22.80	21399.02
341	Mhow	22.55	75.75	589	841.74	1965.30	15.87	20.41	20456.67
342	Midnapore	22.43	87.32	49	890.52	2182.95	18.51	22.22	20183.33
343	Mirajgaon	18.73	75.02	566	606.43	1579.45	17.85	22.41	21168.60
344	Modasa	23.46	73.29	144	909.17	2069.90	17.38	21.28	20110.83
345	Mohana	22.71	75.85	532	1014.93	1997.15	18.17	20.68	19740.83
346	Mokhada	19.93	73.34	393	968.67	2212.60	19.70	22.47	20341.67
347	Molakalmuru	14.71	76.74	607	779.83	2071.70	21.38	24.15	20591.26
348	Morsi	21.32	78.01	349	738.04	1879.70	18.94	22.84	20129.00
349	Morvi	22.81	70.82	43	498.48	1426.90	18.46	23.08	20929.33
350	Mulbangal	13.16	78.39	847	963.76	2200.70	19.29	20.65	19637.69
351	Mungeli	22.06	81.68	288	915.15	2107.55	18.11	21.74	20255.46
352	Murari	25.34	78.43	283	606.93	1579.65	17.97	22.20	20562.07
353	Murtajapur	20.73	77.35	304	607.68	1617.40	18.50	22.85	20668.60
354	Nadiad	22.69	72.86	32	657.38	1618.65	17.74	22.03	20113.33
355	Nagarkarnool	16.49	78.31	448	708.36	1783.40	20.29	23.43	20714.13
356	Nagpur	21.14	79.08	301	901.61	2123.40	17.74	21.03	20416.13
357	Nakhatrana	23.34	69.26	130	478.95	1207.50	17.86	22.08	19865.67
358	Nalesar	20.03	79.28	213	895.86	2208.95	18.61	22.44	21029.17
359	Nalhati	24.29	87.83	32	608.22	1582.35	18.05	22.23	20593.10
360	Nandgaon	18.38	72.92	465	710.49	1817.85	17.15	21.86	21139.22
361	Nandura	20.83	76.45	263	547.24	1571.05	20.92	24.46	20315.58
362	Narajole	22.56	87.61	12	882.89	2199.10	18.69	22.36	20528.33
363	Narasapatam	16.43	81.69	11	593.34	1519.35	21.43	24.04	19640.79
364	Narayanganj	22.28	79.57	590	846.16	1736.25	14.13	18.50	20530.83
365	Narayangarh	24.27	75.05	449	574.52	1316.20	18.06	21.84	19918.92
366	Narayanpur	19.72	81.24	528	899.47	1984.10	16.64	20.57	20915.83
367	Narsingarh	23.71	77.08	517	925.85	1996.75	16.72	20.67	20155.00
368	Narsinhpur	21.1	77.84	333	935.13	2093.40	17.75	21.48	20253.33
369	Navapur	21.16	73.79	132	867.10	2074.00	16.98	21.32	20934.17
370	Navipet	18.77	78.01	360	869.99	2319.05	20.13	23.69	20710.17

371	Neemuch	24.47	74.86	483	885.77	1839.10	15.36	19.36	19623.33
372	Ner	20.49	77.86	333	617.69	1618.15	18.69	22.86	20653.49
373	Nevasa	19.55	74.92	469	713.87	1855.35	18.27	22.59	20906.00
374	Nimbahera	24.62	74.68	440	893.62	1955.15	16.12	20.33	19850.83
375	Nirmal	19.09	78.34	325	641.60	1648.85	19.59	23.22	20766.28
376	Nithawa	23.55	74.18	220	858.24	2129.55	17.38	21.78	20072.50
377	Niwas	23.04	80.44	653	881.23	1642.50	13.95	17.72	19946.67
378	Nizamabad	18.67	78.09	395	876.28	2313.15	20.12	23.64	20684.75
379	Nowgong	25.06	79.43	229	847.03	1779.35	14.43	18.86	19675.83
380	Onda	23.13	87.2	70	909.98	2131.40	18.11	21.80	20109.17
381	Pachhar	24.63	76.72	318	897.89	1823.60	15.17	19.23	19584.17
382	Pachmarhi	22.46	78.43	1067	825.31	1537.55	12.98	16.85	20115.00
383	Pachwara	25.37	79.05	208	867.25	1808.65	14.83	19.11	19586.67
384	Palakonda	18.6	83.75	37	642.80	1690.45	20.89	23.93	20728.24
385	Palamau	24.03	84.07	218	910.61	1811.30	15.19	19.13	19595.00
386	Palanpur	24.17	72.43	222	829.08	2092.40	16.65	21.47	20034.17
387	Palasdanga	24.61	87.8	48	912.09	2126.05	18.10	21.75	20013.33
388	Palhera	22.05	80.76	565	880.26	1983.65	16.43	20.56	20379.17
389	Panderkowra	20.1	78.33	247	611.97	1720.30	20.30	24.05	20516.28
390	Pangree	19.81	74.2	570	672.72	1818.15	18.57	22.79	21009.28
391	Panskura	22.39	87.74	5	746.88	1785.80	18.41	22.08	20290.91
392	Paraswada	22.17	80.29	576	879.11	1806.00	15.15	19.08	20123.33
393	Parbhani	19.26	76.77	412	674.74	1720.50	18.74	22.74	21015.22
394	Partabgarh	25.89	81.94	88	878.75	1922.00	15.95	20.05	19765.00
395	Parvatipuram	18.78	83.42	119	700.45	1903.70	20.89	24.08	20911.58
396	Patan2	23.18	79.42	375	861.40	1935.30	15.90	20.16	20195.83
397	Patrasayar	23.19	87.54	46	750.81	1748.60	18.03	21.70	19981.82
398	Patur	20.46	76.93	335	756.39	1910.20	19.25	22.95	20494.06
399	Peint	20.25	73.5	604	979.41	2005.70	17.47	20.75	20945.00
400	Pendra Road	22.75	81.89	602	926.17	1805.45	15.77	19.08	19778.33
401	Perambalur	11.24	78.86	134	1050.37	1947.25	18.05	20.26	19426.67
402	Pindraon	27	78.65	146	910.69	2209.20	18.81	22.44	20431.67
403	Pinglaj	22.81	72.6	23	833.76	2257.15	18.27	22.84	20510.83
404	Pirawa	24.16	76.03	376	908.33	1924.60	16.05	20.07	19781.67

405	Pithora	21.25	82.51	288	927.79	2087.40	17.76	21.43	20766.67
406	Polur	12.51	79.12	171	649.54	1336.25	18.71	21.41	21224.68
407	Potkabari	23.52	88.27	15	565.27	1255.25	16.94	21.02	19678.38
408	Prantij	23.43	72.85	118	873.62	2112.95	17.30	21.64	20068.33
409	Pudukkottai	10.37	78.82	95	429.87	1151.20	21.93	24.63	20791.07
410	Purnea	25.77	87.47	35	880.70	1886.00	15.81	19.75	19513.33
411	Purulia	23.33	86.36	257	917.55	2015.60	17.27	20.83	19938.33
412	Radhanpur	23.82	71.61	27	686.56	1876.65	18.14	22.81	19777.00
413	Raiganj	25.61	88.12	28	900.82	1934.95	16.42	20.16	19533.33
414	Raipur	21.25	81.62	302	924.21	2125.85	18.21	21.75	20315.00
415	Raipur 2	21.07	78.96	294	866.45	1998.15	18.12	21.72	20448.67
416	Rajakhera	26.89	78.17	155	803.90	1694.25	15.60	19.44	19374.55
417	Rajgarh	24	76.72	370	716.90	1754.70	17.08	21.59	19545.00
418	Rajim	20.96	81.89	276	760.41	1841.80	18.34	22.10	20781.37
419	Rajkot	22.3	70.8	126	857.15	2244.35	18.62	22.74	20592.50
420	Rajnandgaon	21.09	81.03	307	817.42	1888.60	17.81	21.52	20762.96
421	Rajnigiri	21.27	86.46	545	945.51	1749.00	15.37	18.61	19994.17
422	Rajpipla	21.87	73.5	45	438.87	1262.80	19.94	24.11	20403.17
423	Ramachandrapuram	16.83	82.03	10	372.43	1014.80	22.15	24.79	19638.78
424	Ramgarh1	23.38	85.31	335	956.66	1989.40	17.21	20.61	19941.67
425	Rampurhat	24.18	87.78	33	885.95	2117.50	17.91	21.83	19950.42
426	Ramtek	21.39	79.32	319	783.47	1819.00	17.55	21.53	20361.54
427	Ranaghat	23.17	88.56	9	854.70	2167.20	18.17	22.25	20542.02
428	Ranibandh	22.86	86.78	169	902.47	2019.70	17.16	20.86	20274.17
429	Rasra	25.85	83.85	58	577.08	1259.20	15.12	19.14	19203.33
430	Raver	21.24	76.03	229	751.64	1927.45	18.44	22.57	20648.08
431	Rayaguda	18.17	80.63	62	421.06	1117.05	20.83	24.02	20966.07
432	Rehli	23.63	79.06	402	957.87	1948.35	16.80	20.27	20144.17
433	Rewa	24.53	81.3	295	684.78	1419.20	16.17	19.64	19286.81
434	Robertaganj	24.68	83.06	311	878.27	1858.95	15.46	19.52	19408.33
435	Roha	18.43	73.11	9	451.12	1326.90	22.16	25.13	21180.95
436	Roomal	22.31	80.07	368	861.47	1985.25	16.21	20.58	20626.67
437	Rudri	20.66	81.55	321	702.12	1659.35	17.62	21.70	20635.11
438	Sabroom	23	91.71	18	677.02	1574.15	18.64	22.14	20540.23

439	sachor	24.75	71.77	57	843.42	2173.45	17.65	22.15	19922.50
440	Sachour	24.75	71.77	57	843.42	2173.45	17.65	22.15	19922.50
441	Sagar	23.83	78.73	530	957.81	1975.70	17.37	20.50	19605.83
442	Sagardighi	24.29	88.08	28	904.30	2072.90	17.49	21.31	19973.33
443	Sagwara	23.66	74.02	157	492.01	1422.40	18.16	23.02	20258.67
444	Sailana	23.45	74.92	543	448.84	1266.00	20.32	24.16	19712.70
445	Sakoli	21.08	79.99	243	489.18	1314.25	18.58	22.83	21044.29
446	Sakti	22.02	82.96	231	860.62	2163.15	18.91	22.68	20301.72
447	Saleteka	21.61	79.93	275	935.87	2143.00	18.40	21.89	20142.50
448	Sallopat	23.18	74.16	238	857.00	2141.00	17.47	21.88	20231.67
449	Saltora	23.52	86.93	153	728.83	1698.65	18.22	21.92	19668.42
450	Sambalpur	21.46	83.98	155	731.18	1789.80	18.28	22.12	20517.17
451	Sanand	22.99	72.37	34	864.47	2159.50	17.65	22.03	20324.17
452	Sangareddy	17.61	78.08	513	905.31	2258.60	19.95	23.17	20847.46
453	Sangod	24.92	76.28	271	881.41	1991.15	16.36	20.63	19861.67
454	Saoner	21.38	78.92	320	885.05	2209.20	18.54	22.44	20600.83
455	Saranga	23.14	87.75	34	753.11	1723.25	17.83	21.45	20123.23
456	Sarangarh	21.58	83.07	223	775.08	1806.70	17.81	21.58	20763.11
457	Sarathi	14.6	75.81	521	856.72	2152.95	17.82	21.97	20434.17
458	Sardarpur	22.66	74.97	511	718.30	1711.65	18.43	22.06	20205.26
459	Saswad	18.35	74.03	765	750.66	1744.95	16.41	20.82	20840.38
460	Satana	20.59	74.2	559	865.40	2097.85	17.17	21.52	20971.67
461	Sattenapalli	16.39	80.15	70	575.71	1641.30	21.33	24.57	21265.00
462	Satyavedu	13.43	79.96	35	437.75	1197.10	21.91	24.71	21079.31
463	Sausar	21.65	78.79	350	903.81	2137.00	17.99	21.84	20535.83
464	Savli Tank	22.56	73.22	44	692.63	1827.80	17.56	22.32	20250.00
465	Selod	21.66	73.15	26	894.02	2048.75	17.81	21.54	20835.90
466	Seoni	22.08	79.54	602	866.16	2098.10	17.20	21.52	20657.50
467	Serampore	22.74	88.33	6	575.89	1319.30	17.20	21.19	20093.51
468	Shahabad1	30.15	76.86	256	825.26	1813.50	14.56	19.15	19443.33
469	Shahpura	23.18	80.7	648	644.00	1182.50	12.84	16.90	19597.83
470	Shajapur	23.42	76.27	443	902.12	1992.35	16.73	20.64	19936.67
471	Shegaon	20.79	76.69	276	490.87	1272.50	20.87	23.95	19915.63
472	Sheoganj	25.14	73.06	271	939.96	2134.05	18.29	21.82	19439.17

473	Shergarh1 (punjab)	30.08	74.94	198	881.75	2046.10	16.92	21.08	20000.00
474	Sholapur	17.65	75.9	466	428.59	1048.40	20.36	23.49	21087.04
475	Shujalpur	23.38	76.71	448	894.21	2034.90	16.88	20.99	20049.17
476	Sihora	23.48	80.1	384	928.26	1893.40	16.23	19.81	19899.17
477	Silda	22.61	86.81	106	933.73	2058.95	17.65	21.19	20357.50
478	Silli	23.34	85.85	237	925.41	2099.20	18.10	21.53	20035.00
479	Simdega	22.61	84.49	433	931.14	1941.85	16.71	20.22	19930.00
480	Simlapal	22.91	87.07	55	749.61	1744.55	17.93	21.66	20250.51
481	Sindewahi	20.29	79.66	203	879.30	2130.10	18.20	22.09	20946.61
482	Sinnar	19.85	74	680	860.43	2045.45	17.26	21.37	20480.51
483	Sirohi	24.88	72.85	306	910.22	2157.45	18.30	22.01	19456.67
484	Sironj	24.10	77.67	467	913.39	1920.15	16.09	20.03	19665.83
485	Sirpur	21.34	74.87	160	899.67	2111.85	17.63	21.63	20854.17
486	Sirsi	14.61	74.84	608	1043.78	2147.80	19.75	21.93	19640.83
487	Sirur	16.09	75.78	537	505.36	1143.20	18.76	22.21	20715.87
488	Sitamau	24.01	75.35	480	916.88	1885.60	15.83	19.75	19773.33
489	Sohagpur	22.7	78.19	324	866.43	1802.95	14.99	19.06	20160.00
490	Sonamukhi	23.3	87.41	49	755.02	1735.30	17.90	21.57	19964.65
491	Sonamura	23.47	91.26	13	945.27	2175.60	18.63	22.16	20112.50
492	Songadh	21.16	73.56	119	823.22	2383.75	19.81	23.90	20946.67
493	Sonua	22.57	85.46	266	943.50	1939.45	16.66	20.20	20365.00
494	Srikakulam	18.29	83.89	15	599.14	1555.65	21.10	24.00	20182.05
495	Srinivaspur	13.33	78.2	813	1007.24	2156.55	19.18	22.00	20430.00
496	Srungavarapukota	18.11	83.13	87	623.59	1449.80	20.59	23.13	19463.16
497	Surda	22.55	86.44	113	706.71	1723.80	19.80	22.99	19737.36
498	Susner	23.94	76.1	415	934.64	1957.95	16.84	20.35	19787.50
499	Suvasra	24.06	75.65	462	877.35	1874.40	15.50	19.65	19853.33
500	Talcher	20.95	85.21	77	559.49	1439.65	19.08	23.00	20942.11
501	Talegaon	20.4	74.9	385	448.35	1211.90	20.46	23.93	20208.20
502	Talegaon Dhamdhera	18.67	74.14	564	679.03	1673.75	18.16	22.24	20866.30
503	Tallakulam	9.93	78.13	114	597.81	1506.80	21.34	23.88	19632.89
504	Taloda	21.56	74.21	123	462.66	1430.15	20.79	24.78	20285.51
505	Tamar	23.05	85.65	248	937.31	2075.60	17.87	21.33	19986.67

506	Tamia	22.34	78.66	932	959.86	1871.70	16.11	19.63	20188.33
507	Tarakeswar	22.88	88.01	5	855.32	2186.40	18.77	22.56	20541.53
508	Tasgaon	17.02	74.6	577	741.98	1902.85	19.32	23.07	21403.00
509	Telhara	21.02	76.84	270	789.23	2176.85	19.56	23.65	20933.33
510	Tendukhera	23.39	79.53	393	905.45	2174.90	18.34	22.16	20472.50
511	Teonthar	24.98	81.64	96	889.58	1766.40	14.82	18.75	19806.67
512	Tharad	24.03	71.42	10	493.50	1311.40	16.62	21.78	20418.92
513	Tharsa	21.22	79.38	263	903.03	2220.40	18.66	22.54	20945.00
514	Tirora	21.4	79.93	276	786.82	1868.45	18.17	22.00	20441.35
515	Tiruvannamalai	12.22	79.07	168	561.17	1579.50	22.09	24.84	20889.47
516	Tiruvur	17.1	80.6	98	718.98	2107.05	20.94	24.50	21046.60
517	Titlagarh	20.28	83.14	212	751.36	1811.40	17.85	21.80	21160.78
518	Tuni	17.35	82.54	24	378.92	1007.90	22.12	24.65	19244.90
519	Udaipur	24.58	73.71	552	862.08	1793.60	14.71	18.98	19665.83
520	Ujjain	23.17	75.78	489	885.63	1960.35	16.27	20.37	19945.00
521	Uluberia	22.47	88.1	8	905.80	2186.80	18.76	22.26	19896.67
522	Umaria	23.53	80.82	456	901.36	1839.75	15.52	19.36	19903.33
523	Umarkhed	19.59	77.69	416	728.38	1836.25	17.82	22.22	21301.98
524	Umrer	20.96	79.2	315	901.08	2171.80	18.44	22.13	20670.00
525	Unchhera	24.38	80.79	327	899.83	1745.75	14.62	18.58	19765.83
526	Viramgam	23.12	72.05	35	831.63	2278.75	18.48	23.02	20265.83
527	Vishnupur	23.06	78.99	342	911.51	2125.25	18.07	21.74	20105.00
528	Wadhawan	22.42	71.4	76	852.47	2184.65	17.87	22.24	20450.83
529	Waghai	20.77	73.49	140	906.83	2228.85	19.17	22.61	20540.83
530	Wanghroli Tank	22.53	73.19	72	611.62	1621.45	17.73	22.47	20215.91
531	Wani	20.06	78.95	211	441.80	1007.70	19.56	22.74	21140.74
532	Wankaner	22.62	70.95	77	371.24	1069.85	18.23	23.18	21441.07
533	Wara-Main	21.37	80.24	305	909.53	2036.70	17.88	21.44	20296.58
534	Waraseoni	21.76	80.05	305	568.70	1271.45	18.16	21.71	20586.11
535	Wardha	20.74	78.6	274	614.45	1718.70	20.32	24.03	20386.05
536	Washim	20.11	77.13	541	900.30	2070.75	17.31	21.29	20870.00
537	Yaval	21.16	75.69	219	468.41	1149.20	18.26	22.30	20820.63
538	Yelgattor	18.55	78.25	315	850.00	2323.30	20.30	23.89	20664.10
539	Yeotmal	20.23	78.08	456	770.24	1823.00	19.27	22.64	20794.90

540	Yeracaud	11.77	78.2	1407	968.92	2212.65	19.77	22.47	19848.33

Class #4 (Locations: 23)

Sr No	Station	Lat	Long	Alt	P days (7,21,30)	GDD (4 deg C base temp)	Mean Night temp	Mean T	Mean radn (Kj/day/m2)
1	Amari	29.94	78.52	900	623.15	1493.60	17.63	21.21	14217.24
2	Amritsar	31.63	74.87	229	623.15	1493.60	17.63	21.21	14217.24
3	Amritsara	31.63	74.87	229	624.16	1495.25	17.69	21.23	14143.68
4	Amroha	28.90	78.46	217	488.45	1312.90	19.24	22.87	15501.43
5	Buxaduar	26.46	89.35	752	928.68	1653.15	15.06	17.81	14739.17
6	Dharamsala Upper	32.11	76.28	702	691.15	1593.45	17.31	20.81	13845.26
7	Fazilka	30.40	74.03	182	574.45	1514.45	17.46	21.66	15347.67
8	Ferozpore City	30.93	74.62	188	597.00	1486.95	17.39	21.34	15215.12
9	Haflong	25.16	93.01	656	924.55	1664.00	14.95	17.90	14397.50
10	Jaimalwala	30.75	75.04	216	598.44	1478.65	17.27	21.24	15218.60
11	Jalalabada	30.36	74.15	184	583.36	1514.25	17.59	21.65	15251.16
12	Mandangad	17.98	73.26	240	654.28	1282.65	19.29	21.39	15025.68
13	Moga	30.81	75.17	225	596.72	1479.90	17.23	21.25	15259.30
14	Muktesar	30.47	74.51	197	587.33	1496.40	17.37	21.45	15319.77
15	Nagamangala	12.82	76.25	771	798.15	1757.90	21.23	23.15	14432.61
16	Nagode	24.57	80.57	320	848.98	1781.10	14.68	14.25	14938.36
17	Nathana	30.31	75.09	213	585.36	1521.95	17.69	21.74	15344.19
18	Pandavapura	12.49	76.66	728	799.78	1752.30	21.15	23.09	14523.91
19	Raya	29.81	75.11	202	626.37	1507.30	17.91	21.37	13917.24
20	Rupar	31.02	76.58	275	649.01	1607.60	17.80	21.52	14896.74
21	Taran-Taran	31.46	74.92	217	620.40	1490.90	17.57	21.18	14274.71
22	Yellapur	14.96	74.71	555	818.99	1714.90	20.93	22.68	14073.91
23	zirakpur	30.96	74.98	211	598.32	1483.65	17.34	21.30	15275.58

Class #5 (Locations: 17)

Sr No	Station	Lat	Long	Alt	P days (7,21,30)	GDD (4 deg C base temp)	Mean Night temp	Mean T	Mean radn (Kj/day/m2)
1	Ammathi	12.24	75.86	932	1127.02	1935.95	18.87	20.17	7719.17
2	Belur	13.16	75.86	941	1077.77	1912.50	18.58	19.97	7726.67
3	Bhagamandala	12.38	75.52	892	1113.37	1941.15	18.93	20.21	7695.00
4	Chikmagalur	13.32	75.78	1036	1096.84	1972.75	18.85	20.47	6539.17
5	Karike	12.44	75.42	335	1113.88	1916.85	18.50	20.01	7914.17
6	Mahabaleshwar	17.93	73.64	1273	1020.57	1574.15	16.04	17.15	6885.00
7	Mercara	12.42	75.73	1112	1061.40	1710.50	16.93	18.29	7165.00
8	Mukteshwar	29.28	79.39	2311	895.38	1503.35	14.00	10.97	7103.31
9	Murkhal	19.92	79.84	181	1107.22	1933.10	18.73	16.56	7810.96
10	Nagerhole	12.12	76.15	859	748.16	1348.35	19.29	20.70	8055.56
11	Napoklu	12.3	75.68	902	1079.01	1965.50	18.91	20.41	8031.67
12	Pulingoth	12.2	75.34	919	1120.54	1999.40	19.26	20.70	8346.67
13	Saklespur	12.94	75.78	900	1081.90	1954.05	18.77	20.32	7477.50
14	Sanivarasanthi	12.72	75.88	956	1107.61	1949.70	19.02	20.28	7254.17
15	Simla	31.1	77.17	2184	823.65	1316.15	13.34	15.00	7623.33
16	Sunticoppa	12.45	75.82	990	1122.18	1994.75	19.25	20.66	7824.17
17	Virajpet	12.19	75.8	894	1086.64	1788.05	17.73	18.93	7935.83

Class #6 (Locations: 12)

Sr No	Station	Lat	Long	Alt	P days (7,21,30)	GDD (4 deg C base temp)	Mean Night temp	Mean T	Mean radn (Kj/day/m2)
1	Anamalais	10.67	77.00	282	984.53	1606.50	15.63	17.42	9223.33
2	Anantnag	33.73	75.14	1589	701.08	1514.20	13.83	16.65	9484.17
3	Balecove	11.59	76.05	924	1072.96	1908.95	18.32	19.94	9651.67
4	Balehonnur	13.34	75.46	741	1072.96	1908.95	18.32	19.94	9651.67
5	Darjeeling	27.04	88.26	2145	818.51	1306.90	13.28	14.92	9490.00
6	Devala	11.47	76.38	890	1109.63	2027.50	19.25	20.93	10221.67
7	Karmad	19.87	75.54	565	1114.44	2076.45	19.88	21.34	9107.50
8	Koppa	13.53	75.36	765	1101.66	1907.60	18.38	19.93	9664.17
9	Kulgam	33.64	75.02	1742	549.69	1123.10	14.76	17.91	9760.49
10	Periyapatna	12.33	76.09	844	1121.22	2091.20	20.01	21.46	8947.50
11	Shillong	25.57	91.89	1421	916.11	1458.50	14.39	16.19	9508.33
12	Vantipore	33.53	74.54	1600	722.42	1692.45	17.68	20.96	9534.00

Class #7 (Locations: 166)

Sr No	Station	Lat	Long	Alt	P days (7,21,30)	GDD (4 deg C base temp)	Mean Night temp	Mean T	Mean radn (Kj/day/m2)
1	Anantpur	25.11	75.85	295	505.16	1513.95	21.21	24.79	21531.51
2	Anekal	12.71	77.69	913	975.66	2216.80	19.45	22.51	21311.67
3	Armori	20.47	79.98	218	840.77	1977.45	17.32	21.53	21268.14
4	Arsikere	13.31	76.25	802	660.26	1781.95	19.85	23.63	23131.87
5	Attur	11.60	78.60	217	428.85	1326.00	22.34	25.45	22343.55
6	Aundh	18.56	73.81	578	971.88	2102.35	18.25	21.55	21334.17
7	Bagalkot	16.17	75.65	535	659.41	1842.55	20.91	24.29	22136.26
8	Bagepalli	13.78	77.79	725	930.44	2315.95	20.27	23.33	21177.50
9	Bangalore	12.97	77.59	900	902.69	2215.30	19.48	22.81	21189.83
10	Bansgaon	26.54	83.34	68	513.01	1525.15	19.33	23.86	22302.60
11	Baramati	18.18	74.16	564	698.42	1354.85	18.06	22.19	21901.67
12	Barur	12.30	78.30	425	466.50	1277.45	21.16	24.34	22252.38
13	Belgaum	15.84	74.49	770	885.85	2354.40	20.60	23.82	22066.39
14	Bellary	15.13	76.92	447	499.86	1524.15	21.20	24.93	21757.53
15	Bhivandi	19.25	73.09	9	488.89	1438.70	20.91	24.61	21782.86
16	Bhongir	17.50	78.88	404	856.71	2235.30	19.96	23.47	21480.87
17	Bhopalpatnam	18.86	80.38	142	715.15	2110.35	20.69	24.53	21388.35
18	Bhor	18.14	73.84	621	885.85	2354.40	20.60	23.82	22066.39
19	Bijapur	16.83	75.71	609	772.65	1890.65	18.71	22.39	21662.14
20	Bijawar	24.62	79.49	342	772.65	1890.65	18.71	22.39	21662.14
21	Bolthan	20.19	74.10	586	884.49	2156.25	17.85	22.00	21208.33
22	Brahmapuri	24.85	74.60	398	835.83	2016.30	17.68	21.88	21209.73
23	Challakire	14.31	76.65	585	462.19	1275.20	20.96	24.30	22284.13
24	Chandgad	15.95	74.19	702	948.64	2222.20	19.36	22.55	22731.67
25	Chittor	13.22	79.10	306	707.61	1844.05	20.90	23.87	22056.99

26	Chowsala	18.71	75.69	696	882.93	2198.85	18.21	22.36	21741.67
27	Cuddahpah	14.47	78.82	124	376.83	1264.95	22.39	25.88	22141.38
28	Dahivadi	17.70	74.54	732	913.93	2304.00	19.81	23.23	21719.17
29	Davangire	14.47	75.92	588	904.59	2349.65	20.47	23.61	21296.67
30	Denkanikottah	12.53	77.79	863	738.64	1870.30	19.91	23.32	22493.81
31	Devanhalli	13.24	77.71	903	889.51	2243.95	19.68	23.05	21346.61
32	Dhamtari	20.70	81.55	308	786.93	1928.15	17.78	21.89	21224.07
33	Dharamapuri	12.11	78.14	471	497.17	1488.65	21.62	25.02	22318.31
34	Dharampur	20.54	73.18	79	560.18	1520.25	19.50	23.54	21639.74
35	Dharwar	15.46	75.01	728	847.59	2303.05	19.05	23.23	22772.50
36	Dhone	15.40	77.87	421	546.50	1668.10	21.28	24.90	22065.00
37	Dodballapur	13.29	77.54	911	884.95	2258.60	19.80	23.17	21328.81
38	Dohad	21.81	75.94	270	884.95	2258.60	19.80	23.17	21328.81
39	Gadag	15.43	75.64	656	830.60	2293.00	20.37	23.97	21659.13
40	Gaganbavada	16.54	73.83	614	637.47	1293.65	18.82	21.54	21516.22
41	Gandhinlaj	16.23	74.35	642	419.40	1267.25	20.25	24.50	22393.55
42	Goribindnur	13.61	77.52	684	942.76	2284.35	19.97	23.07	21304.17
43	Gudiband	13.67	77.70	827	944.21	2253.70	19.65	22.81	21239.17
44	Gulabgarh	30.58	76.86	298	838.29	1609.50	14.36	17.45	10929.17
45	Hadagalli	15.02	75.93	520	397.83	1197.85	20.67	24.72	22503.45
46	Haliyar	15.20	74.45	550	580.41	1481.10	19.84	23.29	22645.45
47	Hanamkonda	18.01	79.56	265	729.66	1993.25	20.00	23.77	21365.35
48	Hangal	14.77	75.13	586	849.48	2368.60	20.66	24.11	22644.07
49	Harur	12.05	78.48	338	634.97	1847.40	21.67	24.80	21658.43
50	Haveri	14.80	75.40	571	717.66	1886.75	19.86	23.49	22420.62
51	Hindupur	13.82	77.50	629	421.89	1201.75	20.27	24.03	22478.33
52	Hiriyur	13.94	76.62	596	565.82	1552.75	20.84	24.22	22312.99
53	Holakhere	14.02	76.11	692	577.18	1499.50	19.90	23.53	22364.94
54	Honnali	14.23	75.65	529	579.26	1483.40	19.69	23.32	22346.75

55	Hosadurga	13.80	76.29	709	887.66	2245.70	19.77	23.23	22312.82
56	Hosur	12.74	77.83	856	982.05	2132.25	18.81	21.80	21541.67
57	Hubli	15.36	75.12	616	898.04	2345.25	20.35	23.58	21597.50
58	Hudugur	13.64	77.57	685	633.10	1509.25	19.50	22.68	23159.26
59	Indapur	18.11	75.03	508	855.62	2276.20	18.77	23.00	21841.67
60	Islampur	26.25	88.20	55	886.48	2341.95	20.02	23.55	21255.00
61	Jagdalpur	19.07	82.01	559	930.54	2071.80	17.55	21.30	21148.33
62	Jamkhed	18.74	75.31	591	387.98	1050.75	17.34	22.50	21940.35
63	Jath	17.05	75.21	660	881.90	2204.20	19.55	23.04	21480.17
64	Kadur	13.55	76.01	765	900.25	2222.05	19.60	23.03	22404.27
65	Kalghatgi	15.19	74.97	557	664.66	1826.65	20.71	24.12	22448.35
66	Kalyan	19.24	73.13	6	521.25	1658.00	21.73	25.31	21616.67
67	Kanakapura	12.55	77.42	625	847.61	2272.15	20.33	23.79	22256.52
68	Kapashi	16.33	74.28	565	683.90	1871.50	19.92	23.74	21904.21
69	Karad	17.28	74.20	572	871.20	2338.70	20.08	23.69	22343.70
70	Karjat	18.92	73.33	47	785.27	2304.15	21.17	24.61	21933.04
71	Khairee	24.78	79.82	241	841.39	1978.45	17.34	21.54	21265.49
72	Khandala	20.03	74.77	581	575.66	1397.30	18.32	22.20	21737.66
73	Khed	17.72	73.40	18	509.03	1424.35	17.81	22.79	22381.58
74	Kolar	13.14	78.13	837	949.89	2244.90	19.62	22.74	21333.33
75	Kolhapur	16.70	74.24	561	823.52	2174.50	19.34	23.28	21905.31
76	Kollegal	12.15	77.11	649	711.87	1919.15	20.49	23.83	21677.32
77	Koratagere	13.52	77.24	742	725.52	1903.45	20.18	23.66	21907.22
78	Koregaon	17.70	74.16	662	913.75	2301.20	19.82	23.21	22341.67
79	Krishnarajnagar	12.44	76.38	810	973.81	2212.55	19.65	22.47	21130.00
80	Kudligi	14.91	76.39	586	511.54	1512.15	21.18	24.77	21957.53
81	Kuppam	12.75	78.33	691	543.10	1287.90	20.50	23.28	21307.46
82	Kurnool	15.83	78.04	284	377.10	1162.30	21.44	25.21	22025.45
83	Kurud	20.83	81.72	300	838.42	2144.35	18.21	22.36	21400.00

84	Kurundwad	16.68	74.59	543	352.90	984.40	20.34	24.17	21961.22
85	Lonavala	18.75	73.41	630	1012.45	1963.75	17.60	20.40	21236.67
86	Madakasira	13.94	77.27	665	699.03	1881.85	20.34	23.85	22329.47
87	Maddikera	15.25	77.42	461	517.99	1488.10	20.76	24.44	22053.42
88	Maddur	12.58	77.04	633	463.07	1294.90	20.71	24.30	22851.56
89	Madha	18.03	75.52	488	777.95	2165.40	19.31	23.54	21909.01
90	Magadi	12.96	77.23	854	894.32	2250.90	19.87	23.11	21327.12
91	Malavalli	12.38	77.04	622	964.74	2239.45	19.72	22.70	21690.00
92	Malur	13.00	77.94	905	890.84	2236.05	19.60	22.98	21408.47
93	Medchal	17.63	78.48	576	914.10	2236.95	19.10	22.67	21239.17
94	Medha	17.79	73.83	710	1006.17	2222.50	19.94	22.55	21333.33
95	Miraj	16.81	74.64	553	443.88	1277.25	19.78	24.02	22190.63
96	Mudhol	16.33	75.28	544	457.72	1287.95	20.40	24.19	22268.75
97	Mudigere	13.13	75.64	960	1027.02	2080.00	18.67	21.37	22281.67
98	Mundargi	15.2	75.88	524	849.45	2251.65	20.03	23.61	21625.22
99	Murbad	19.26	73.38	61	530.17	1520.30	20.71	24.32	21524.00
100	Mysore	12.29	76.63	733	688.56	1758.85	19.97	23.37	22467.03
101	Nalgonda	17.05	79.26	375	466.78	1236.00	20.08	23.68	21990.48
102	Nanded	19.13	77.32	351	803.46	2142.25	19.36	23.34	21481.08
103	Nanjangud	12.11	76.68	649	564.97	1422.15	20.26	23.54	22184.93
104	Narasinharajapura	13.6	75.5	688	938.13	2283.30	19.92	23.06	22939.17
105	Nargund	15.72	75.38	603	722.35	1973.90	19.70	23.58	22508.91
106	Navalgund	15.56	75.35	575	548.11	1478.60	19.95	23.77	22312.00
107	Ne lamangala	13.08	77.41	866	894.97	2219.50	19.50	22.84	21191.53
108	Niphad	20.07	74.1	554	855.50	2088.60	16.91	21.44	21457.50
109	Osmanabad	18.1	76.03	655	737.31	1916.60	18.61	22.83	21730.39
110	Palacode	12.29	78.07	491	451.36	1328.80	21.35	24.83	22934.38
111	Palliwada Pro	17.2	79.18	263	625.75	1817.00	20.87	24.46	21555.06
112	Panvel	18.98	73.11	12	471.22	1354.15	21.21	24.58	21637.88

113	Paud	18.52	73.61	579	597.83	1337.95	17.77	21.43	21844.16
114	Peddapalli	18.61	79.38	228	851.99	2326.05	19.79	23.58	21389.08
115	Pennagaram	12.13	77.89	496	637.88	1819.40	21.11	24.49	22264.04
116	Periyanaickampalayam	11.15	76.93	431	695.09	1783.45	20.50	23.64	21550.55
117	Phaltan	17.98	74.43	554	534.42	1417.60	19.45	23.47	21926.03
118	Pimpalner	20.94	74.12	517	492.86	1371.45	17.26	22.34	21726.67
119	Pune	18.52	73.85	559	911.06	2183.00	18.43	22.23	21592.50
120	Punganur	13.36	78.57	720	966.32	2176.25	19.01	22.17	21266.67
121	Pusad	19.91	77.56	335	863.29	2327.30	19.45	23.43	21302.50
122	Pusesauali	17.46	74.31	716	917.02	2290.35	19.64	23.12	21833.33
123	Puttur1	13.43	79.55	145	445.93	1171.25	21.27	24.26	21687.93
124	Radhanagari	16.41	73.99	564	720.04	1565.30	19.10	22.04	21967.82
125	Raibag	16.49	74.77	571	403.59	999.50	19.95	23.30	21936.54
126	Raichur	16.21	77.34	388	548.52	1591.55	21.31	24.72	21641.56
127	Ramanagaram	12.7	77.28	668	943.36	2291.55	20.11	23.13	21808.33
128	Ramdurg	15.95	75.29	569	622.50	1814.90	20.73	24.44	22200.00
129	Ranebennur	14.61	75.63	577	624.01	1597.60	19.78	23.30	22583.13
130	Risod	19.97	76.78	530	452.38	1355.60	18.99	23.70	21560.87
131	Sangamner	19.57	74.2	559	485.19	1435.35	18.02	23.19	21912.00
132	Sangli	16.85	74.58	565	524.97	1340.45	19.89	23.48	21843.48
133	Satara	17.68	74.01	687	1027.46	2159.60	19.41	22.03	21278.33
134	Sendamangalam	11.28	78.23	179	428.44	1200.55	21.73	24.77	22651.72
135	Shahapur	19.45	73.32	54	388.28	1058.55	20.45	24.05	21641.51
136	Shevgaon	19.35	75.21	481	856.26	2333.75	19.59	23.48	21230.83
137	Shiggaon	14.99	75.22	610	655.49	1767.65	19.95	23.69	22921.11
138	Shirala	16.98	74.12	593	650.42	1720.45	19.51	23.38	22010.11
139	Shirol	16.74	74.59	551	556.25	1409.05	19.76	23.36	21747.95
140	Shrigonda	18.61	74.69	550	871.93	2127.60	17.50	21.76	21916.67
141	Siddapur	14.34	74.88	565	1003.90	2179.15	19.37	22.19	22755.83

142	Somawarpet	12.59	75.85	1100	1021.09	1916.70	17.32	20.01	22541.67
143	Sorab	14.38	75.1	591	865.83	2357.90	20.33	23.85	23031.09
144	Sringeri	13.41	75.25	632	987.10	2251.05	20.05	22.79	22497.50
145	Srirangapatna	12.42	76.69	667	581.43	1470.35	20.08	23.40	22618.42
146	Sukma	18.39	81.65	192	646.50	1711.05	19.37	23.27	21688.76
147	Tarikere	13.7	75.81	663	571.33	1366.75	19.24	22.78	22834.25
148	Thathaiengarpet	11.12	78.44	134	453.13	1162.80	21.21	24.12	21684.48
149	Tiptur	13.26	76.47	845	477.16	1248.25	19.93	23.57	22389.06
150	Tiruchengode	11.37	77.89	242	375.84	1235.65	22.30	25.75	23222.81
151	Tiruppattur	10.1	78.59	94	411.55	1217.35	21.41	24.63	21806.78
152	Tiruttani	13.17	79.61	109	417.31	1215.75	21.87	25.03	22500.00
153	Trimbak	19.93	73.53	709	979.61	1962.45	17.12	20.39	21193.33
154	Tumkur	13.33	77.11	818	913.22	2201.40	19.51	22.85	21757.26
155	Turuvekere	13.16	76.66	785	661.88	1781.80	19.87	23.62	23189.01
156	Uppilyapuram	11.26	78.51	170	508.26	1246.95	20.62	23.55	22229.69
157	Uttangarai	12.26	78.53	327	405.63	1223.35	21.43	24.73	21654.24
158	Vada	19.65	73.14	55	468.55	1287.20	20.64	24.18	21490.63
159	Vadgaon	18.73	73.63	631	880.05	2132.95	17.68	21.81	21651.67
160	Vainiyambadi	12.69	78.62	335	415.48	1210.65	21.29	24.52	21664.41
161	Vellore	12.91	79.13	214	487.81	1298.30	21.49	24.35	21551.56
162	Wai	17.94	73.89	697	1001.74	2236.50	20.07	22.67	21315.83
163	Wallajah	12.91	79.35	154	478.83	1317.75	21.86	24.65	21500.00
164	Warora	20.24	79.01	203	877.13	1959.85	16.66	20.79	21316.24
165	Yadki	15.03	77.52	301	372.11	1177.85	21.86	25.49	22236.36
166	Yeola	20.04	74.48	560	894.55	2165.50	18.07	22.08	21077.50

Class #8 (Locations: 15)

Sr No	Station	Lat	Long	Alt	P days (7,21,30)	GDD (4 deg C base temp)	Mean Night temp	Mean T	Mean radn (Kj/day/m2)
1	Aryankavu	8.98	77.15	255.00	434.6183866	928.35	21.40153061	23.02755102	12095.91837
2	Dharamsala Lower	32.11	76.28	702.00	1004.657664	2031.3	18.96416667	20.96083333	13129.16667
3	Gundlupet	11.81	76.69	782.00	1048.381303	2131.25	19.88354167	21.79375	12865
4	Hamirpur	31.69	76.52	763.00	866.1490415	1747.3	16.57675439	19.3622807	12540.35088
5	Jammu	32.73	74.86	307.00	677.2372816	1461.85	16.66929348	19.93315217	13161.95652
6	Jorhat	26.75	94.20	78	986.6651224	1805.75	16.02979167	19.08125	13177.56113
7	Kalyani	22.98	88.43	9	877.7916656	2151.15	17.814375	21.95958333	13115.61622
8	Matherau	18.98	73.27	760	1090.862357	2136.9	20.105	21.84083333	13283.33333
9	Muhammadpur	28.41	76.98	219	876.4839088	1742.6	14.7025	13.09764706	12474.70588
10	Mustafabad	28.71	77.26	212	874.5087444	1717.85	14.70395833	11.71196809	12018.61702
11	Muzaffarpur	26.12	85.36	52	905.3169734	1886.65	16.03770833	14.2810241	13683.13253
12	Pantnagar	29.02	79.49	222	877.7916656	2151.15	17.814375	21.95958333	13115.61622
13	Pasighat	28.06	95.32	157	894.6672436	1769.25	16.925	19.69247788	13312.38938
14	Supa	20.99	73.03	17	410.0275438	823.15	20.66833333	22.38111111	12782.22222
15	Talavadi	21.66	74.51	199	798.7607304	1745	21.04673913	23.01086957	12930.43478

Class #9 (Locations: 16)

Sr No	Station	Lat	Long	Alt	P days (7,21,30)	GDD (4 deg C base temp)	Mean Night temp	Mean T	Mean radn (Kj/day/m2)
1	Banjar	31.38	77.20	1520.00	1027.576	2142.6	19.88875	21.88833	11961.66667
2	Berinag	29.77	80.05	1703.00	771.3936	1423.5	14.82762	17.59524	12533.33333
3	Gulabgarh	30.58	76.86	298.00	838.292	1609.5	14.35625	17.44583	10929.16667
4	Holenarsipur	12.78	76.24	836.00	838.9473	1664.1	20.51413	22.13152	10752.17391
5	Hunsur	12.30	76.29	770.00	830.2219	1699.7	20.8337	22.51848	10978.26087
6	Kausani	29.84	79.60	1682.00	890.0802	1463.25	14.04396	16.22708	10679.16667
7	Kotgarh	31.31	77.47	1928.00	1062.275	1775.5	17.15375	18.82917	10301.66667
8	Kotkhai	31.12	77.54	1721.00	1054.032	1960.45	18.73104	20.37042	12263.33333
9	Kulu	31.96	77.11	1213.00	853.4055	1461.05	13.64813	16.19458	12195
10	Kurseong	26.88	88.28	1543.00	980.6337	1765.6	16.1625	18.74667	10731.66667
11	Multai	21.77	78.25	753	923.7822	1837.25	15.86271	10.79651	11156.27907
12	Nagar	31.21	77.07	1174	718.8786	1189.95	11.25938	12.13007	11633.33333
13	Nagina	29.44	78.43	247	875.4053	1713.35	14.85521	11.04196	10750.75377
14	Pedong	27.15	88.61	1427	1108.601	2046.6	19.67083	21.08833	10527.5
15	Tamenglong	24.98	93.49	1149	1074.09	1902.8	17.99083	19.89	11185.83333
16	Ukhrul Farm	25.18	94.13	1349	1021.071	1735.75	16.41229	18.49792	11541.66667

Class #10 (Locations: 7)

Sr No	Station	Lat	Long	Alt	P days (7,21,30)	GDD (4 deg C base temp)	Mean Night temp	Mean T	Mean radn (Kj/day/m2)
1	Bironkhol	29.84	79.03	1498.00	838.2441804	1577.75	14.60520833	17.18125	12997.5
2	Champawat	29.34	80.09	1652.00	770.4644458	1493.4	15.22904762	18.26095238	13766.66667
3	Kalimpong	27.06	88.47	1183.00	970.9777629	1594.75	15.855625	17.32291667	14159.16667
4	Kasauli	30.90	76.96	1821.00	693.7627591	1435.25	14.54684466	17.97330097	13274.75728
5	Mawsynram	25.29	91.58	1425	877.4291563	1369.6	13.14541667	15.44666667	14179.16667
6	Nowshera	34.07	74.81	1588	545.5868564	1325.55	18.11166667	21.78066667	13897.33333
7	Srinagar (J & K)	34.08	74.80	1579	601.2770632	1036.35	12.02201087	15.30815217	13691.37243

Class #11 (Locations: 3)

Sr No	Station	Lat	Long	Alt	P days (7,21,30)	GDD (4 deg C base temp)	Mean Night temp	Mean T	Mean radn (Kj/day/m2)
1	Chakarata	30.70	77.87	2127.00	853.4438525	1489.7	15.23108108	17.45495495	15527.92793
2	Dalhousie	32.54	75.97	2022.00	941.4498978	1918	17.27958333	20.01666667	14908.33333
3	Pauri	30.14	78.77	1689	927.2204712	1690.35	15.84979167	18.11958333	15130.83333

Class #12 (Location: 1)

Sr No	Station	Lat	Long	Alt	P days (7,21,30)	GDD (4 deg C base temp)	Mean Night temp	Mean T	Mean radn (Kj/day/m2)
**1**	Glenmorgan	11.50	76.59	1959.00	836.5583785	1341.8	12.72291667	15.215	22594.16667

Class #13 (Locations: 5)

Sr No	Station	Lat	Long	Alt	P days (7,21,30)	GDD (4 deg C base temp)	Mean Night temp	Mean T	Mean radn (Kj/day/m2)
1	Heggaddevankote	12.09	76.32	701.00	569.9531343	1540.6	20.34615385	23.8025641	24055.12821
2	Jalandhar	31.33	75.58	233	700.3162133	1664.9	13.24416667	17.9075	24968.03727
3	Krishnarajpet	12.66	76.49	790.00	654.0795531	1715.6	20.42643678	23.76551724	24312.64368
4	Pimpalgaon	20.16	73.98	571	1116.494112	2353.75	20.100625	24.34791667	24450.83333
5	Vaduj	17.59	74.45	734	505.2555919	1482.4	20.05608108	24.08648649	24429.72973

Class #14 (Locations: 2)

Sr No	Station	Lat	Long	Alt	P days (7,21,30)	GDD (4 deg C base temp)	Mean Night temp	Mean T	Mean radn (Kj/day/m2)
**1**	Kodaikanal	10.24	77.49	2079.00	871.6834278	1426.85	13.27520833	15.92375	19412.5
**2**	Ootacamund	11.4	76.69	2237	918.3219638	1544.85	14.19479167	16.90708333	19005

Class #15 (Locations: 2)

Sr No	Station	Lat	Long	Alt	P days (7,21,30)	GDD (4 deg C base temp)	Mean Night temp	Mean T	Mean radn (Kj/day/m2)
**1**	Mussoorie	30.45	78.06	1984	938.9359072	1986.55	17.51104167	11.43773148	6219.907407
**2**	Nainital	29.38	79.46	1941	851.5831129	1487.25	13.94770833	7.328066914	5084.386617
